# Termination of psychotherapy: a systematic review

**DOI:** 10.1080/28324765.2025.2535626

**Published:** 2025-07-22

**Authors:** Yocheved L. Rabinowitz, Brian Yim, J. Christopher Muran

**Affiliations:** Derner School of Psychology, Adelphi University, Hy Weinberg Center, Garden City, NY, USA

**Keywords:** Adult individual psychotherapy termination, systematic review, PRISMA, Termination

## Abstract

This paper provides a systematic review of literature on the termination phase of adult individual psychotherapy, adhering meticulously to the PRISMA guidelines. The review was carried out independently by two researchers. The initial search results yielded 1809 articles, although after exclusion criteria, 48 articles were identified. Backward citation added 19 relevant articles, resulting in the retrieval of 67 articles. Data of the retrieved articles were extracted and reported, covering various themes and ideas such as reasons for termination, types of terminations, the initiation of termination, duration of therapy, termination criteria, termination discussion, the termination period, patients’ feelings at termination, therapists’ feelings at termination, processing termination, successful or unsuccessful terminations and post-termination contact. The synthesis of this empirically based data serves as a valuable resource for both researchers and clinicians, facilitating easy access to important information.

## Termination of psychotherapy: a systematic Review

Regardless of treatment length, modality, setting or orientation, psychotherapy termination is an inevitable and critical process for all dyads (O’Donohue & Cucciare, 2010). The termination phase of therapy raises numerous questions regarding its implementation and process. For instance, who initiates the termination, and on what criteria is that decision based? In cases of disagreement within the dyad, how is termination navigated? What is the appropriate duration for termination, and does it vary between short- and long-term treatments? How is a therapeutically effective conclusion characterized? What emotions does termination elicit for both therapists and patients, and how are these addressed post-termination? Does all communication between the therapist and patient cease after termination, and how far in advance should termination be discussed before the final session? Under what circumstances, through what means and for what reasons might the termination process be expedited? Despite the myriad of questions and concerns regarding termination, many articles, journals, books and publications published about termination reiterate surprise about the lack of research on this phase of psychotherapy (Fragkiadaki & Strauss, 2011; Gelso & Woodhouse, 2002; Knox et al., 2011; Schachter et al., 2018; Weil et al., 2017; Roe, Dekel, Harel, & Fennig, 2006).

Historically, the literature has focused on theoretical contributions from a primarily psychoanalytic perspective based on long-term treatments. Analysts have postulated and theorized various facets of termination, encompassing considerations of when to conclude therapy and the feasibility of a full or meaningful ending (Råbu, Binder, et al., 2013). However, even the theoretical literature is scarce, and Schachter et al. (2018) writes that analysts have resisted studying or thinking about termination. Novick (1982) sees this resistance as failure to point out and acknowledge mismanagement of termination by psychoanalytic pioneers and past errors. He also asserts that therapists may deny any strong feelings and be unwilling to talk about post-termination contact (Schachter et al., 2018).

Freud seems not to have believed in the possibility of a completed and successful analysis, but rather saw termination as an abrupt and negative ending to the impossibility of deep, productive, therapeutic work (Goldberg, 1975). By contrast, Levinson (1977) posits that a good ending can influence whether the patient’s gains continue to be made after the ending of treatment, and Schlesinger (2005) writes that “ending is what therapy is all about”. Zinkin (1994) points out that there is no agreed-upon way of when or how to end therapy, and Joyce et al. (2006) conclude that “termination must be tailored to the specific patient and treatment and warrants some degree of attention in all treatments” (pg. 14). Evolving from Freud’s view on termination, numerous preceding viewpoints emphasize the profound significance of the concluding phase of therapy, each offering distinct perspectives.

In cognitive behavioral therapies (CBT) there is a recognized belief that the duration of therapy can significantly influence the pace of change, and dropout from treatment often occurs without a clear plan for termination (Wheeler, 2013). Consequently, CBT practitioners typically initiate discussions about termination with patients from the outset of therapy. During these discussions, patients are encouraged to articulate their treatment goals, evaluate their progress and estimate the duration they anticipate being in therapy (Vidair et al., 2017; Wheeler, 2013). Therapists in this modality emphasize the goal-oriented nature of CBT and its time-limited framework. Patients are educated about the non-linear nature of progress and the possibility of setbacks even after treatment concludes, while also being reassured that they will acquire skills to manage challenges independently over time (Vidair et al., 2017). Throughout the course of therapy, therapists in CBT frequently remind patients about the planned termination to facilitate their preparation and processing of the ending (Wheeler, 2013).

Additionally, a key aspect of termination in CBT revolves around the quality of the therapeutic alliance. A strong therapeutic alliance not only aids patients in acquiring the necessary skills but also fosters self-efficacy, ultimately preparing them to become their own “therapists” (Vidair et al., 2017). Typically, termination in CBT is gradual, with sessions spaced out over time—starting from bi-weekly sessions and gradually extending to monthly, quarterly and semi-annual meetings—to address any necessary modifications or changes and ensure sustained success (Wheeler, 2013).

Analytic contributions have stressed the pain of loss, mourning and abandonment, seeing termination as a dark and difficult phase (Gelso & Woodhouse, 2002), giving minimal consideration or excluding entirely, its more positive aspects such as gratitude (Gelso & Woodhouse, 2002; Roe, Dekel, Harel, Fennig, & Fennig, 2006). More recent empirical contributions have looked at patients’ and therapists’ feelings associated with termination (Roe, Dekel, Harel, Fennig, & Fennig, 2006), uncovering and highlighting these positive aspects.

Empirical studies have also looked at factors related to the termination process, such as what is important to discuss, as well as who initiates the process (Gelso & Woodhouse, 2002; Joyce et al., 2006; O’Donohue & Cucciare, 2010; Strupp & Binder, 1985). Several studies differentiate patient-initiated unilateral termination from the processes that occur in agreed-upon psychotherapy termination and indicate that terminations that were initiated by therapists and/or agreed upon terminations were more likely to be associated with higher treatment satisfaction and better outcomes (Olivera et al., 2017; Swift & Greenberg, 2012).

Successful terminations are repeatedly shown to correlate with better treatment outcomes. In fact, from therapists’ perspectives, empirical data shows that a successful termination phase is associated with better overall treatment outcomes (Bhatia & Gelso, 2017). Furthermore, from the patients’ perspectives, positive terminations impacted them positively and provided confidence for future coping, while conversely, negative terminations lead to a decline in the therapeutic relationship (Knox et al., 2011). These viewpoints underscore the importance of focusing on proper termination making this phase of therapy a focal point for knowledge and research.

While empirical investigations into the termination phase of treatment remain sparse, existing efforts continuously point to the importance of this phase of psychotherapy for treatment to be considered successful from both the patient and therapist perspectives. The slow emergence of empirical data highlights the importance of focusing on this phase of therapy.

## Specific aims

The aim of this project is to provide a systematic review of empirical studies in adult individual psychotherapy terminations based on the PRISMA guidelines. Although numerous specific and important research questions can be raised concerning termination, this review is guided by the topics and themes identified within the existing body of published research with a focus on learning what is already known about termination. Since the literature on psychotherapy termination is scant, such a review can open the door for future research that can address the gaps. It can also aid clinicians to learn about termination, so they can address it clinically in a way that is productive and helpful to patients. A meta-analysis was not undertaken due to the substantial heterogeneity in methodology within this field. This project uses the word termination focusing specifically on the sessions leading up to and including the final session between dyads in individual psychotherapy.

## Method

### Identification of studies

To synthesize the existing literature on termination, a systematic review adhering to the Preferred Reporting Items for Systematic Reviews and Meta-Analyses (PRISMA) guidelines was conducted. These guidelines offer standardized methods for researchers to identify, select, assess and synthesize studies in systematic reviews (Moher, 2009). By rigorously following these guidelines, the study ensures a meticulous and transparent approach, enhancing the reliability and validity of the synthesized information.

The electronic databases Google Scholar and PsychArticles were searched using the keywords “adult individual psychotherapy termination” and “termination of psychotherapy” excluding “premature termination”. The electronic database PsychInfo was searched with those same terms; however, due to the large number of search results yielded, additional filters were applied. The filters included age group above 18 years old as well as empirical studies. All initial search results from the three searches (up to 10 January 2024) were documented and organized in an Excel spreadsheet in the order retrieved, with duplicates manually identified and removed. A reference management program was not used.

Two independent researchers read the titles and abstracts to eliminate the papers that did not follow the inclusion/exclusion criteria as well as any duplicate results that emerged. The eligibility of each article was decided based on the title and abstract, however, when those two did not fully determine eligibility, the body of the article was examined as well. All included articles were reviewed and agreed upon by both researchers. When uncertainty remained, those articles were discussed with the research director and eligibility was determined via consensus. The research director has over 35 years of expertise in research methodology and data analysis; their role focused on guiding the research process rather than providing content expertise in psychotherapy termination. Finally, backward citation searches were conducted of the articles obtained to identify any potentially relevant articles that may have been missed in the electronic searches.

### Selection criteria

Articles included in the review were based on the following inclusion/exclusion criteria: All empirically based peer-reviewed research on adult individual termination written in English was included to ensure methodological rigor and quality; unpublished data were excluded as it can limit the replicability and reliability of findings. Excluded articles were non-systematic case studies which often have a large overlap with theory: termination with specific groups, such as child, group or family therapy; termination with specific populations, such as termination with borderline personality disorder patients or eating disorder patients; theoretical papers; unpublished dissertations that have not undergone the peer-review process; terminations that are not related to clinical psychotherapy, such as termination in the medical/biology sciences; and lastly, premature termination.

### Quality assessment

Each article underwent assessment by two independent reviewers to establish eligibility. Although several quality assessment tools including the United States National Institutes of Health (National Institute of Health NIH, 2021), Newcastle-Ottawa Scale (NOS), Joanna Briggs Institute (JBI) and Mixed Method Appraisal Tool (MMAT) were considered, research suggests that conducting such evaluations may be futile. Critics contend that these assessment tools are inadequate for capturing the thoroughness of a study, particularly in the case of qualitative research (see Levitt et al., 2025). They argue that a peer-reviewed article’s publication already implies a certain level of quality assessment. Furthermore, it is rare for an article to be excluded from a review solely based on the results of these assessment tools. As such, none of the assessment tools considered were used.

## Results

**Figure 1.**
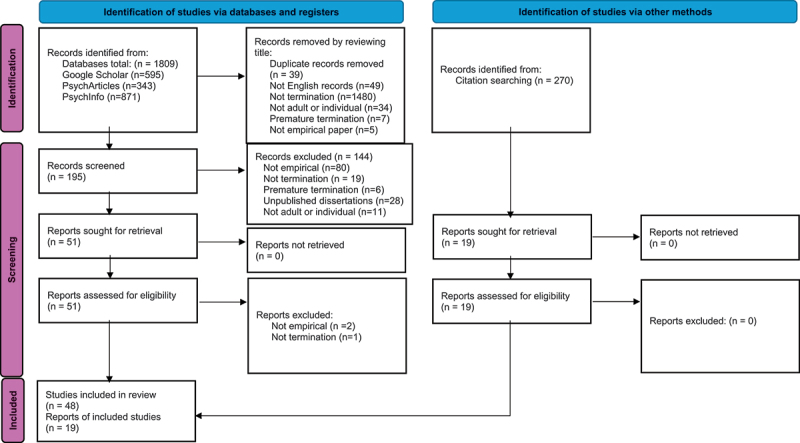
PRISMA 2020 flow diagram for new systematic reviews which included searches of databases, registers and other sources.

The Google Scholar search yielded 595 results, the APA PsychArticles search yielded 343 results and the APA PsychInfo search yielded 871 results. All original 1,809 titles were reviewed for initial exclusion by two researchers. Of those articles, 1480 were not about termination of psychotherapy, 49 were not written in English, 39 were duplicates, 7 were premature termination, 5 were not empirical articles, and 34 were not adult or individual termination. The remaining 195 articles were screened by using the abstract and body of the article, whereby 144 were excluded. Records excluded 80 not empirical papers, 19 not about termination of psychotherapy, 6 that were premature termination, 11 that were not adult or individual termination, and 28 were unpublished dissertations. The remaining 51 articles were retrieved and assessed for eligibility. Thirty-eight of those were agreed upon by the first and second author and 13 were reviewed with the third author and agreed upon via consensus. Two of those were not empirical and one was not about termination. The remaining 48 articles were included. Backward citation searches yielded additional 19 results and reviewed by both researchers for consensus. Total included articles were 67. See PRISMA chart (Figure 1) for details of included articles. Some studies that addressed both premature termination and other aspects of termination were included; although when not applicable, the premature results were omitted from the review.

From: Page MJ, McKenzie JE, Bossuyt PM, Boutron I, Hoffmann TC, Mulrow CD, et al. The PRISMA 2020 statement: an updated guideline for reporting systematic reviews. BMJ 2021;372:n71. do i: 10 .1136/bmj.n71. For more information, visit: http://www.prisma-statement.org/.

### Data extraction

Of all identified articles, study design, sample size and characteristics, assessed variables, intervention and intervention results were extracted. The extracted data was synthesized in a table format (Table S1 presented as supplemental material). In addition, the results of each study were synthesized and summarized narratively by termination topics.

## Review of studies

### Reasons for termination

Therapy termination occurs for various reasons, with the most common being external factors and goal achievement. Reported rates of termination due to external factors vary significantly across studies. From patients’ perspectives, the prevalence ranges from 28% (Olivera et al., 2017) to 54.6% (Roe, Dekel, Harel, & Fennig, 2006) and approximately 60% (Cooke et al., 2020), with other studies reporting rates as low as 11% and as high as 87%. Therapists reported termination due to external reasons at rates from 11.5% to 40.2%, with some variation based on professional roles or levels of training (e.g., De Bosset & Styrsky, 1986; Pollak et al., 1992; Bhatia & Gelso, 2017). Combined perspectives generally echoed this variability, with clinic records showing a termination rate of 19.9% due to external factors (Renk & Dinger, 2002). Several studies mention external reasons as a factor in termination without specifying percentages (e.g., A. E. Fortune, 1985; Knox et al., 2011).

Termination due to goal achievement also exhibited variability, with rates reported by patients ranging from 40% to 53%, while therapists’ estimates were notably lower, from 11.5% to 27%. Studies incorporating both perspectives (Todd et al., 2003) revealed significant discrepancies; for example, Hunsley et al. (1999) reported 25.8% from therapists’ perspectives versus 44% from patients’. Partial goal achievement was more frequently acknowledged by patients, highlighting differences in perceptions (Cuffel et al., 2000). While some studies did not provide specific percentages, they identified goal completion as a meaningful factor in therapy termination (A. E. Fortune, 1985; Knox et al., 2011).

Beyond external factors and goal achievement, many other reasons for therapy termination are documented. Dissatisfaction with therapy or the therapist is frequently cited by patients. For instance, 25% reported dissatisfaction in Olivera et al. (2017), with additional rates of 36.4% dissatisfaction with the therapist and 29.9% dissatisfaction with therapy in Roe, Dekel, Harel, and Fennig (2006). Hynan (1990) reported 39% discomfort with services, while Hunsley et al. (1999) found that 34% cited therapy “going nowhere”, 30% reported therapy misalignment with their idea of treatment, 30% lacked confidence in the therapist, and 16% felt uncomfortable talking to the therapist. Other patient-reported reasons included losing interest in therapy (15%) or discomfort with the therapy process (7.2%; Westmacott & Hunsley, 2010). Dissatisfaction, while a prominent theme, was sometimes referenced without exact percentages, encompassing issues such as unmet expectations, poor communication, therapy mismatches and perceptions that therapy made things worse (Cooke et al., 2020).

Therapists also reported various practical and dynamic reasons for termination. Practical reasons included excessive caseloads (11%; DeBerry & Baskin, 1989), referral to another service (6%) and experiencing an impasse in treatment (3.1%; De Bosset & Styrsky, 1986). Dynamic factors included fear-based responses, such as fear of dependency (37%), fear of intimacy (30%), fear of abandonment (19%) and negative transference (16%; Pollak et al., 1992). Additionally, therapists occasionally noted dissatisfaction with therapy as a reason for termination (34%; Pollak et al., 1992).

Other reasons arose from records and combined analyses. Some patients terminated without notice or reason (35%; Renk & Dinger, 2002), while others found treatment elsewhere (11.5%). Specific cases of therapy “running its course” (11%), feeling they had nothing to talk about (19%), needing independence (14.3%) or engaging in new relationships (11.7%) were also reported (a, 2006). Similarly, logistical barriers (4.1%), illness (4.1%) and therapy being perceived as too painful (6.3%) were observed in cases from Mosher-Ashley (1994) and other records. Late termination often correlated with significant improvements, while patients who felt therapy ended prematurely cited financial constraints, mismatches or inadequate therapist containment (Roe, 2007).

Patients and therapists often differed in their perspectives on termination. For example, patients were more likely to attribute termination to external factors, while therapists cited goal achievement as a stronger reason. Studies such as those by Hunsley et al. (1999) indicate low concordance between patient and therapist reports, though others, like Westmacott et al. (2010), suggest higher agreement in mutual terminations. These findings underscore the complexity of therapy termination and the need for further investigation to understand its dynamics more comprehensively.

Research has yet to fully elucidate the factors influencing these discrepancies and the broader variability in termination reasons. Exploring the interplay between patient and therapist perspectives, as well as the dynamics of mutual versus unilateral terminations, could provide valuable insights into the processes and outcomes associated with therapy conclusion.

### Types of terminations

The literature identifies several types of therapy terminations: mutual, unilateral, forced, transfer and premature. Mutual termination occurs when both therapist and patient agree to end therapy, while unilateral termination typically involves the patient ending therapy without notice or with minimal warning. Forced termination happens when the therapist is compelled to terminate, often due to external circumstances. Transfer termination involves the therapist leaving and transferring the patient to another clinician, which can overlap with forced termination but may also be planned. Premature termination refers to patients leaving therapy before the designated time or without fully engaging, often overlapping with unilateral terminations. This review focuses on mutual, unilateral and forced terminations, excluding premature terminations due to their unique features and prevalence in research. Pollak et al. (1992) found 66% of terminations were premature, 73% were not negotiated, 71% were anticipated, and only 27% negotiated. In this study, 41% were premature yet anticipated, 29% were unexpected, and 28% were unrelated to therapy. Gelman (2009) reported that social work students encountered 24% natural terminations, 35% patient-initiated terminations, and 93% forced terminations. Corning and Malofeeva (2004) found in a university setting that 34.5% terminated prematurely, 31% mutually and 34.5% due to external factors like graduation.

Termination patterns vary by type and session progression. Premature terminations decline over time, whereas mutual terminations are less frequent at the start, increase until around session 8, plateau until approximately session 28, and then exhibit a subsequent increase(Corning & Malofeeva, 2004). Tryon & Kane (1993) found that unilateral terminators typically had fewer sessions compared to mutual terminators. Prior therapy experience reduces the likelihood of mutual and premature terminations, while canceled sessions lower the chances of mutual terminations, and no-shows increase the likelihood of premature terminations. Patients referred to therapy by others are more likely to terminate prematurely or mutually. Younger individuals making progress in therapy often experience mutual terminations, while therapy modality does not significantly affect termination type (Edbrooke-Childs et al., 2021; Fragkiadaki & Strauss, 2011; Keleher et al., 2019). High alliance ratings are consistently linked to mutual and planned terminations Tryon & Kane (1995), while unilateral terminations often result from ruptures in the therapeutic relationship (Knox et al., 2011; Westmacott et al., 2010). Connell et al. (2006) reported unilateral terminations were common before the third session, often due to lack of motivation. Later, unilateral terminations occurred when goals were achieved or symptoms improved. Roe (2007) found 40% treatments ended on time, 37% early and 23% late, with satisfaction lower in early and late terminations. Gelman (2009) noted natural terminations correlated with ease and satisfaction, while patient-initiated terminations evoked frustration and lack of closure.

### Transfer terminations

Transfer terminations are most successful when the departing therapist demonstrates nurturing, mature qualities and good documentation practices and emphasizes empathy, genuineness and responsibility. Conversely, reserved or passive behavior, poor billing practices or inadequate processing of the loss can hinder the process. The new therapist’s emotional responses also play a crucial role; patience, acceptance and skill in managing negative patient affect facilitate transitions, while insecurity, anxiety about rejection or inexperience can hinder it. Patient factors, including their pathology, preferences and expectations, significantly impact the transition, and clinic-provided structure, such as facilitating meetings between the old and new therapist, can ease the process (Marmarosh et al., 2017).

Residents often struggle to inform patients about their departure, delaying disclosure and experiencing emotions such as guilt, anxiety, sadness and occasionally relief (Schen et al., 2013). Departing clinicians may feel uneasy addressing patients’ positive feelings, while new therapists must navigate comparisons to their predecessor and address the patient’s negative emotions about the transfer. The absence of a meeting between therapists can increase patient anxiety. Schulman and Kay (1989) explored cases where clinicians transferred patients to their new practices, typically for continuity of care. While some supervisors endorsed these transfers, others viewed them as sensitive transference/countertransference dynamics, necessitating careful consideration and supervision.

### Termination initiation

Research indicates that patients typically initiate therapy termination, often without mutual agreement. Kramer (1986) and Råbu and Haavind (2018) noted that patients often feel responsible for raising termination, and subtle hints from patients can complicate identifying the initiator (Råbu, Binder, et al., 2013). Bhatia and Gelso (2017) found 66.1% of terminations were mutually agreed upon, while 6.9% were patient-initiated, 1.3% therapist-initiated and 24.5% due to external factors. In Cuffel et al. (2000), 24.7% patients and 3.2% providers reported initiating termination, with most patients (78.9%) claiming it was their own decision. Similar patterns emerged in other studies, with patient-initiated terminations ranging from 59% to 67% (Mosher-Ashley, 1994; Olivera et al., 2013, 2017), though some settings, such as social work training, showed higher rates of therapist-initiated terminations due to placement limits (Baum, 2005).

Patients who initiated termination often exhibited severing behaviors like tardiness and avoiding discussions of loss, while those whose termination was therapist-initiated or due to external reasons expressed greater interest in continuing (Baum, 2005). Therapists’ responses to patient-initiated termination varied, with 56% opposing termination in Roe’s (2007) study and mixed feelings reported by psychiatry residents (De Bosset & Styrsky, 1986). Agreement on termination also varied across studies, with patients agreeing more frequently than therapists (Olivera et al., 2013). Setting differences further influenced termination patterns; for example, patients in community settings more often terminated independently compared to those in nursing home facilities (Mosher-Ashley, 1994).

### Duration of therapy

Research on therapy duration and termination reveals patterns and influencing factors. Studies show that many patients terminate early, with 66% ending therapy before the 50th session (De Bosset & Styrsky, 1986), while others gradually discontinue, with 45% remaining after 12 sessions and only 29% beyond 40 sessions (Pollak et al., 1992). In private practice, 13% terminated unilaterally before the third session, and 67% ended through mutual agreement (Westmacott & Hunsley, 2017). Therapists often highlight building a strong alliance as essential for retention, with unilateral terminators attending significantly fewer sessions (Westmacott et al., 2010).

Patients who terminated before 30 sessions often had therapists rated as less confident (Vaslamatzis et al., 1989). Mutual terminations following successful outcomes tended to involve more sessions than unilateral or externally influenced terminations (Renk & Dinger, 2002). Treatment outcomes, particularly improvement, were strong predictors of session length, especially for patients completing therapy (Goldenberg, 2002). Interestingly, patient expectations for longer treatment predicted lower satisfaction with outcomes Owen et al. (2009), with flexibility and therapist involvement in setting end dates seen as beneficial (Ling & Stathopoulou, 2020).

Psychodynamic perspectives regard therapy as ongoing regardless of session count (Fragkiadaki & Strauss, 2011), while some patients, particularly those with severe or chronic conditions, remain in therapy indefinitely due to barriers like life crises, reliance on therapy or attachment to the therapeutic relationship (Friedlander et al., 2014). Research also emphasizes the role of therapist qualities in patient retention; experienced, female or highly empathetic therapists tend to retain more patients (McNair et al., 1963).

Studies on brief psychotherapy suggest patients with higher suitability scores are more likely to complete the protocol and find it effective (Vaslamatzis et al., 1989). Additionally, patient demographics, therapist training and presenting complaints showed limited correlation with therapy length, though depression was commonly reported among those remaining longer (Pollak et al., 1992). End-date adherence is mixed; 41% of cases extend therapy due to incomplete goals, patient ambivalence or regression. Final decisions to extend or terminate typically hinge on goal achievement, with external factors like caseload and therapist-patient dynamics playing a lesser role (A. E. Fortune, 1985).

### Termination criteria

The theoretical literature explores numerous criteria for termination, yet the evidence-based literature remains notably limited. Kramer (1986) suggests that therapists should actively listen for cues signaling the conclusion of therapy, considering it a termination criterion. In the study by Råbu, Haavind, et al. (2013), the termination of therapy was not straightforward or guided by predetermined criteria but emerged through mutual discussion. The dyads frequently employed structural elements such as temporary breaks or session tapering while processing and planning for termination. Discussions often included the possibility of resuming sessions. Both therapists and patients in this study acknowledged being driven by emotional reactions, some of which were not fully addressed and discussed during the termination process.

### When was termination discussed?

Research emphasizes the importance of early and ongoing termination discussions throughout therapy. Kramer (1986) and Fragkiadaki and Strauss (2011) both highlighted the need for collaboration between therapists and patients regarding treatment conclusion from the start. Trainees often feel a desire to manage termination properly but are concerned about patients’ negative responses (Baum, 2006). Gould (1977) found that half of therapists informed patients about termination within the first three sessions, with timing influenced by supervision. Brill and Nahmani (1993) observed that most trainees initiated termination discussions 4–8 weeks before therapy ended. Interestingly, a study by Harari and Waehler (1999) found no correlation between patients’ perceptions of therapists and whether termination was introduced at the initial session. Gelman (2009) found that while termination was discussed in class, supervision discussions were less frequent, with most students feeling moderately prepared to manage the process.

### Length of termination period

According to De Bosset and Styrsky (1986), planned terminations involved approximately 8% of the therapy duration dedicated to termination discussions, whereas other termination types had minimal to no time allocated for processing termination. Bhatia and Gelso (2017) reported that therapists, on average, spent 16.82% of the therapy duration on termination-related activities. However, they acknowledged a potential underestimation, particularly for therapists uncertain about the treatment duration, where the minimum reported sessions were used to calculate the average.

### Patients’ feelings at termination

Research on patients’ reactions to termination reveals a broad range of emotions, from grief and anger to acceptance and pride. Studies noted that patients tended to express more positive emotions than negative ones during the termination phase (Quintana & Holahan, 1992; Fortune et al., 1992). However, many patients, including those in long-term psychoanalytic treatment, expressed a sense of loss associated with the unique therapeutic relationship, though the intensity varied based on treatment duration and outcomes (Craige, 2002). Positive emotions, such as pride and accomplishment, were often linked to patients achieving their therapeutic goals (Baum, 2005; Marx & Gelso, 1987). Patients who viewed the termination as successful often felt proud, independent and relieved, while those who felt unready or dissatisfied reported greater sorrow, self-doubt and frustration (Baum, 2005). Studies suggest that forced or premature terminations, whether initiated by the therapist or due to external circumstances, typically led to more negative emotional responses, including regret and a perceived need for continued treatment (Roe, Dekel, Harel, Fennig, & Fennig, 2006). Therapist-initiate terminations, in particular, were associated with heightened feelings of hurt and anger, although both self-doubt and sorrow were prevalent among patients regardless of who initiated the termination (Baum, 2005). Furthermore, therapists observed similar mixed emotional responses in their patients, from pride to feelings of regression or anxiety (Gelman, 2009), highlighting the emotional complexities surrounding the conclusion of therapy. While some patients chose to end therapy independently, their decisions were often clouded by feelings of ambivalence or self-doubt, particularly when unresolved issues remained (Råbu & Haavind, 2018). Overall, patients’ emotional responses to termination were closely tied to the therapeutic relationship’s perceived success and the degree of personal accomplishment they experienced.

### Therapists’ feelings at termination

Therapists’ emotional responses to termination vary, influenced by their personal history of loss Boyer & Hoffman (1993), the therapeutic bond (Rabu & Haavind, 2012) and whether they or the patient initiated the end of treatment. Many therapists report feelings of pride and accomplishment in patients’ success Fortune (1987), but also experience sadness, anxiety and doubt, especially when the relationship was strong (Baum, 2007; A. Fortune et al., 1992; Fragkiadaki & Strauss, 2011). Forced or untimely terminations, particularly for student therapists, evoke emotions like anger, frustration and guilt, with students often expressing anxiety about unfinished work and concerns for their patients’ future (Baum, 2006; Gelman, 2009). Therapists tend to experience greater self-doubt and negative feelings when patients initiate termination, in contrast to when they have more control over the process, which leads to more positive emotions and professional satisfaction (Baum, 2007). Termination can also stir reflections on personal loss, mortality and professional competence, with therapists noting that an abrupt end to treatment creates more emotional strain, while mutual terminations tend to feel more fulfilling (Brady et al., 1996; Greene, 1980). Female clinicians, especially those not analytically trained, tend to experience more anxiety and seek to shift to a less formal relationship at termination, while analytically oriented therapists typically express more positive emotions and conclude sessions with greater finality (Greene, 1980). Overall, the therapeutic relationship’s success and the sense of professional achievement play a crucial role in therapists’ emotional responses to termination.

### How was termination processed?

Studies on termination processes highlight the importance of addressing both therapist and patient emotions. The collaborative nature of the conclusion is critical, with participants emphasizing the need for time to process termination, especially in the presence of a history of loss (Fragkiadaki & Strauss, 2011; Marx & Gelso, 1987). Therapists commonly engage in activities such as reviewing therapy, discussing growth and acknowledging the therapeutic relationship (Norcross et al., 2017). However, opinions vary on post-termination behaviors, though a general consensus exists against highlighting the rarity of complete success or using travel metaphors (Norcross et al., 2017).

Video recordings of sessions show that metaphors focusing on accomplishments and future challenges are central to discussions during termination (Råbu, Haavind, et al., 2013). While the emphasis shifts from retrospective evaluation to forward-looking perspectives, not all patients engage equally. In cases where the patient feels the therapy was unsuccessful, termination behaviors tend to be more guarded, with patients avoiding discussions about the end (Baum, 2005; Shafran et al., 2020).

Patients with severe diagnoses, especially those with depression, require more structured termination processes, including review of gains, setting goals and providing advance notice (Fair & Bressler, 1992; Sullivan et al., 2018). Additionally, the quality of the therapeutic relationship plays a crucial role in how termination unfolds, as patients with stronger alliances are more likely to engage in termination discussions (Baum, 2005). In general, preemptively addressing termination leads to better patient outcomes and promotes feelings of satisfaction. Student therapists often express a need for earlier, more supportive conversations on the subject (Gelman, 2009) andtherapists with personal boundary difficulties, not shown with experienced therapists, may experience heightened anxiety surrounding termination and attempt to establish a closer psychological connection with their patients (Greene & Geller, 1980).

### Successful versus unsuccessful terminations

Patient involvement in termination correlates with perceptions of successful therapy (Weil et al., 2017). Successful terminations typically involve active engagement and thorough review, while unsuccessful ones lack closure and emotional discussion, with patients often devaluing therapy. Unsuccessful outcomes can be influenced by factors such as patient dissatisfaction and therapist behaviors, such as relocation or self-interest in therapy (Brady et al., 1996).

Factors like gender, age and access to treatment impact termination success, though treatment costs and diagnosis do not (Safarzade et al., 2018). Ghosting by therapists—discontinuing communication without notice—leads to patient frustration, anxiety and resentment. Patients often attribute ghosting to personal issues or their own behavior but believe their therapist is at fault, with emotions fading over time (Farber et al., 2022).

### Post-termination contact

Therapists often believe their work continues to influence patients after termination and may express curiosity about their patients’ post-therapy lives (Fragkiadaki & Strauss, 2011). Both therapists and patients show a desire for post-termination contact (Gelman, 2009), with some therapists leaving an open-door policy. Studies indicate that many therapists maintain this policy, with up to 78% offering it (De Bosset & Styrsky, 1986), and patients who are satisfied with therapy are more likely to express a willingness to return (Knox et al., 2011; Olivera et al., 2017).

A significant portion of therapists acknowledges having post-termination contact, with 25% initiating it themselves (Jofen-Miller & Fiori, 2017). Two-thirds of patients recontact their therapist within 3 years, mainly to address termination issues. Contact types vary, including brief visits, phone calls and letters, and are more common among women (Hartlaub et al., 1986).

Training for post-termination contact exists, with recent graduates more likely to have received it and established related policies (Jofen-Miller & Fiori, 2017). Therapists generally anticipate positive outcomes from post-termination contact, with more experienced clinicians seeing fewer negative consequences (Jofen-Miller & Fiori, 2017).

### Discussion and limitations

This systematic review of the termination phase of individual psychotherapy elucidates pivotal components integral to the psychotherapeutic process. The findings underscore multifaceted reasons driving psychotherapy termination, the two main ones being external impediments and goal attainment as primary motivators. Notably, patients introduced another factor—dissatisfaction with either the therapy itself or the therapist, an aspect not typically articulated by clinicians. The literature reveals a spectrum of termination types, encompassing mutual, unilateral, forced, transfer and premature terminations. Each termination type elicits distinct responses from both patients and therapists, characterized by unique attributes such as alliance ratings. The duration of attended sessions emerged as closely intertwined with therapist characteristics, including adept alliance-building and inner self-confidence. Furthermore, patient attributes, such as the degree of their problems and their self-critical tendencies, also exhibit an influential role in determining the duration of therapy. Intriguingly, when termination decisions were predetermined, close to half of the cases deviated from the planned conclusion. The timing and duration of termination discussions within the therapeutic dyad proved variable, lacking clear empirical evidence. Both patients and therapists articulate a myriad of emotions linked to termination, with variations depending on whether it was mutual, unilateral, premature or forced. Additionally, the quality of the therapeutic relationship emerges as a significant determinant shaping the emotional landscape surrounding termination discussions. Discussion about termination encompassed reflections on the collaborative endeavors undertaken during therapy together as well as strategic discussions pertaining to the future.

These findings shed light on aspects of termination that are well established, areas that lack clarity and aspects that still require further exploration. For example, the substantial percentage difference found for the length of the termination period raises questions, emphasizing the need for further research to explore and understand optimal time allocation for the termination phase. In the theoretical literature, which is primarily analytic, it is hypothesized that the termination phase tends to be long, from 6 months to 1 year or about 17% of the total number of sessions (Gelso & Woodhouse, 2002), 2 years (Firestein, 1978), or 12% of the total treatment length (Tryon, 2002); however, empirical evidence supporting or refuting this hypothesis is lacking. Furthermore, only two empirically based articles address this aspect of termination, highlighting the imperative for future research on the duration of the termination phase of therapy.

Patients provided reasons for termination due to external constraints, with percentages ranging widely from 7% to 87%. This substantial variation is noteworthy. In contrast, therapist reports for this reason showed less divergence, falling within the range of 13% to 35%. Notably, studies with larger patient samples tended to report lower percentages, while smaller sample studies often yielded higher percentages. This observed difference suggests an area for future research to explore, investigating whether the impact of external constraints diminishes with a larger inclusion of patients.

The emotions linked to termination underscored distinctions between short-term and long-term treatment experiences, as well as variations across termination types. The theoretical discourse predominantly emphasizes the theme of loss, particularly the loss of a significant attachment figure (Firestein, 1974; Garcia-Lawson & Lane, 1997). This review aligns with theoretical literature in the context of termination within an in-depth, long-term psychoanalytic treatment framework, as evidenced by Craige (2002). However, the applicability of these theories weakens when applied to shorter or less comprehensive therapeutic modalities. Possibly, generalizations based on theoretical frameworks should be grounded in the intended scope of the theory or substantiated by empirical research.

The existing empirically based literature reveals significant gaps,for example, in the context of long-term treatments. The phenomenon of patients initiating termination prompts the need for in-depth exploration into the reasons behind this trend and strategies for addressing it. Despite extensive theoretical discussions on when to terminate therapy, there is a scarcity of literature documenting the actual implementation of termination criteria, leaving a crucial aspect unexplored. Additionally, topics such as ghosting and post-termination contact are inadequately covered in the current body of literature, highlighting the need for further research and exploration in these areas, while the termination caused by the sudden death of a therapist, despite it likely being a small percentage of terminations, is not researched in the evidenced-based literature. Future research would also benefit from comparing how different therapeutic approaches, such as cognitive-behavioral, psychodynamic and integrative therapies, manage the termination phase and whether distinct patterns emerge across modalities. Similarly, some studies lack detailed information about therapists’ backgrounds and types of therapy offered, which could influence therapy termination outcomes. Research suggests that a variety of therapy approaches may be more effective, and future studies should explore how therapist experience and therapy type impact therapy success and termination. Additionally, examining termination experiences among neurodiverse populations could provide valuable insights, as individuals with autism spectrum disorder or ADHD, for example, may face unique challenges and needs during the conclusion of therapy. Finally, this study included articles from all publication years. Notably, much of the empirical literature was published more than a decade ago, highlighting the need for more current research on termination.

This study stands out for its thorough and comprehensive data collection, presenting the results in a detailed and exhaustive manner. The paper encompasses all available evidenced-based data currently existing on the termination phase of therapy. However, a limitation of this review lies in the diverse participant groups, ranging from students or trainees to experienced clinicians. While Table S1 delineates these distinctions, the results are amalgamated. It would be intriguing to explore whether varying levels of experience among therapists yield distinct results. Additionally, the research settings span clinics, schools, private practices, hospitals, nursing homes, etc. Investigating whether these diverse settings yield different outcomes could be an avenue for future exploration as well. The exclusion of non-English language studies may have limited the scope of the review and should be considered when interpreting the findings. Lastly, individuals with borderline personality disorder, who often experience particular challenges with endings and separation, were excluded from this review. Future research could usefully explore termination processes specifically within this population.

Although each article underwent independent review by two researchers to establish eligibility, no formal quality assessment tool (e.g., NIH, NOS, JBI or MMAT) was used. This decision was based on concerns in the literature that such tools may inadequately capture the methodological rigor of qualitative studies (Levitt et al., 2025) and that peer-reviewed publication already implies a level of quality control. Nevertheless, the absence of a formal quality appraisal remains a limitation of this review and may affect the evaluation of methodological strengths and weaknesses across studies.

While extensive research exists on various aspects of psychotherapy practice, including outcomes, therapeutic alliances and rupture-and-repair dynamics, there is a noticeable dearth of studies focusing on the termination phase underscoring the necessity for future research. Additionally, a systematic review of non-empirical articles on termination can foster new insights, raise questions, deepen understanding and inspire future research ideas.

The findings of this research carry significant implications for both clinical practice and further research in psychotherapy. In practice, understanding the dynamics and factors influencing successful termination can guide therapists in managing the conclusion of therapeutic relationships more effectively. Clinicians can implement evidence-based practices gleaned from the research to enhance the quality of therapeutic endings, ensuring that patients experience a positive and meaningful conclusion to their psychotherapeutic journey. This, in turn, contributes to sustained mental health improvements for patients.

The insights from the systematic review also have implications for ongoing research. Identifying gaps in the current literature on the termination phase prompts further investigation into specific aspects that may be underexplored. Future research endeavors can build upon the findings, delving deeper into the complexities of termination to refine therapeutic practices and contribute to the overall advancement of the field.

In summary, this systematic review provides a comprehensive synthesis of the existing empirical literature on the termination phase of individual psychotherapy. By identifying key reasons for termination, types of termination, emotional responses and the factors influencing the process, the review fulfills its aim of elucidating the complexities of ending therapy. The findings highlight both well-established knowledge and significant gaps, underscoring the need for further empirical research in this critical yet underexplored area of clinical practice. Enhancing our understanding of termination processes holds important implications for improving therapeutic outcomes, guiding clinical training and shaping future research directions.

## Supplementary Material

Supplemental Material

## Public health significance statement

The termination phase of therapy is consistently identified as a neglected aspect of psychotherapy research. In addition, the available data emphasizes the critical importance of this phase for the overall success of the therapeutic process. This systematic review sheds light on the existing information while underscoring the gaps that require attention to ensure the success of the termination phase and, by extension, the psychotherapy process.
