# Supplementary material for: Termination of psychotherapy: a systematic review
Source: Cogent Ment Health. 2025 Jul 22;4(1):2535626. doi: 10.1080/28324765.2025.2535626 (PMC12442594; doi:10.1080/28324765.2025.2535626)
Supplement: Supplemental Material [file OAMH_A_2535626_SM6530.pdf]

Results Table 1. Summary of Studies on Termination

Citation	Sample & Characteristics	Study Design	Main Relevant Findings
Baum N (2005)	Patients: The patients ranged in age from 5 to 84, 66-68% were women and 32-34% were men.<br><br>Therapy ranged from 6 to 99 weeks.<br><br>Therapists: 132 social work students: 92 BSW & 40 MSW, 93% female, BSW students ages ranged from 21-58 and MSW students ages ranged from 24 to 58.<br><br>Setting: Municipal social services including geriatric departments, youth departments, or general welfare departments.	Dependent variables: Emotional Responses to Treatment Termination & Behavioral Responses to Treatment Termination<br><br>Independent variables: Source of the Termination, Speed of the Termination, Sense of Control, Centrality of Therapeutic Relationship, Choice, Desire, Goals Reached, Failure of Therapy<br><br>*All results reported from therapists' perspectives	Who terminated? BSW students: 7.7% patient, 38% therapist, 14.1% jointly, and 40.2% resulted from external factors such as the end of the academic year.<br><br>MSW social workers: 12.5% patient, 42.5% therapist, 32.5% jointly, and 12.5% from external reasons<br><br>There was no difference of feelings expressed by patients from those who worked with the BSW students or those that worked with the MSW students.<br><br>Patients who initiated termination had lower positive feelings, less satisfaction, optimism, pride, sense of success, hope, and happiness. While therapists-initiated patients felt stronger hurt and anger. Self-doubt and sorrow did not differ for patients based on who initiated the termination. However, the less therapy goals were reached and the more they felt they had failed, the more they reported feelings of self-doubt & sorrow.<br><br>Patients who initiated termination showed higher levels of severing behaviors, such as arriving late to session and low discussion of loss experiences. Terminations that were initiated by therapists or terminated for external reasons showed higher patients' interest in continuing.<br><br>Forced terminations, whether circumstantial or initiated by therapist, manifested greater new loss experiences and greater need to continue treatment than those terminations initiated by the client. This group also showed more feelings of self-doubt and sorrow as well as goals not being achieved.<br><br>Patients that wanted to end treatment expressed many more positive self-feelings related to termination and also felt that their goals had been reached.<br><br>Patients who expressed more positive self-feelings at termination correlated highly with a positive therapeutic relationship, attaining their therapeutic goals, believed less that therapy had failed, and was more interested in the termination.<br><br>Patients who tended to be silent around termination showed that the relationship was less central to them, the more they believed therapy had failed, and the more they wanted termination. Those that engaged in discussion

			around termination tended to have more gradual terminations, a strong therapeutic alliance, and believed therapy was successful. They also showed a greater interest in continuing therapy.
Baum N (2006)	Patients: No information provided.<br><br>Therapists: 76 social work students, 42 were in their 1st year in the program and 34 were in the 2nd year in the program. Ages ranged from 25 – 34, majority were female,<br><br>Setting: Either at a municipal social-welfare bureau, a government rehabilitation center, or a general hospital.	Questionnaire distributed asking about their experience of termination.	Responses indicated that termination was untimely (this was a field work placement) and as such feelings expressed: anger that the system forced them to terminate, sadness, regret, frustration at the unfinished work, anxious and uncertain about the quality of their work, guilt, feeling that abandoned client.<br><br>Regarding talking about termination with patients, students felt worried about bringing it up, anticipated negative response from client, and had a desire to terminate properly.<br><br>Post termination: 1/10 expressed desire for continued contact with patients.
Baum N (2007)	Patients: The patients ranged in age from 5 to 84, 66-68% were women and 32-34% were men.<br><br>Therapists: 132 social work students: 92 BSW & 40 MSW, 93% female, BSW students ranged from 21-53 and MSW students ranged from 24 to 58.<br><br>Therapy ranged from 6 to 99 weeks.<br><br>Setting: Outpatient mental health settings.	Dependent variables: Difficulty of Termination, Emotional Valence, and Emotional Responses.<br><br>Independent variables: Source of the Termination.	Who terminated? BSW students: 7.7% patient, 38% therapist, 14.1% jointly, and 40.2% resulted from external factors such as the end of the academic year.<br><br>MSW social workers: 12.5% patient, 42.5% therapist, 32.5% jointly, and 12.5% from external reasons<br><br>Therapists had different feelings when they initiated the termination or when it was mutual to those terminations where the client initiated it. In the latter therapists reported greater self-doubt and lower positive self-feelings. However, their hurt and anger was the same as the other terminations.<br><br>Therapists who reported that the client had terminated treatment showed more negative emotional valence than the others. Moreover, although they did not differ from the other three groups in their hurt/anger, their positive self-feelings were significantly lower, and their self-doubt/sorrow was significantly higher. In contrast, the greater their sense of choice in the termination, the more positive the therapists' emotional valence and self-feelings.<br><br>The more positive the relationship felt to therapists, the more challenging they found termination, the greater their emotional valence and self-feeling, as well as greater hurt and anger.<br><br>When patients initiated terminations, therapists reported higher sense of failure and less positive emotions.

			Therapists tend to find termination moderately difficult.<br><br>The feeling that the goals of the therapy had been attained and that the therapy was not a failure went a long way to making therapists feel satisfaction, optimism, and pride, as well as success, hope, and happiness. This finding highlights the great importance of a sense a professional achievement to therapists' self-feelings at treatment termination.<br><br>Abrupt terminations were reported to be more difficult than gradual ones and to involve less positive self-feelings and more hurt and anger.
Bhatia & Gelso (2017)	Patients: 98 male, 134 female, and 1 other. Ages ranged from 18 to 91. 201 were White, 9 were African American/Back, 10 Hispanic/Latino, 5 Asian/Pacific Islander, and 5 Other.<br><br>Therapists: 233 licensed psychologists identifying a termination phase in recent treatments. 54.5% male, 44.8% female, 94.8% White, 1.7% Hispanic/Latino, 1.7% Asian/Pacific Islander, .9% African American/Black, 1.3% Other. Years of clinical experience ranged from 3 to 65. Theoretical orientations was highly diverse among the sample.<br><br>Setting: Not specified; therapists were recruited through two divisions of the APA.	Working Alliance Inventory – Short Form (WAI-S), Real Relationship Inventory Therapist Form – Shortened (RRI-T), Therapy Session Checklist – Transference Items (TSC-TI), Perceived Client Sensitivity to Loss, Session Evaluation Scale (SES), Counseling Outcome Measure (COM), questions about the termination phase of treatment such as total number of sessions and to indicate what led to patients termination such as therapist's decision, patient's decision, mutual agreement, external factors, or other.	Study conducted from the therapists' perspectives:<br><br>On average, 16.82% of total number of sessions were spent on termination.<br><br>Why terminated? 66.1% mutual agreement, 24.5% external factors, 6.9% client decision, 1.3% therapist decision, 1.3% other.<br><br>From therapist perspective, a successful termination phase is associated with better overall treatment outcomes.<br><br>Therapists that perceive patients who have a greater sense of loss experiences are more likely to identify stronger positive and negative transference during termination.
Boyer & Hoffman (1993)	Patients: Patients seen 69% private practice, 13% clinics, and 18% other.	Therapist termination questionnaire (TTQ), Texas Revised	Counselor loss history and perceived client sensitivity to loss were predictive of counselor affective reactions to termination. Affective reactions analyzed were anxiety and depression.

	Therapists: 117 counselors who treated patients at least 25 sessions and had a termination phase: 61 men and 56 women, 96.6% White, licensed to practice 3-40 years, 49% psychodynamic, 26% CBT, 11% eclectic, 14% client-centered.<br><br>Setting: Not specified; therapists were recruited via the APA.	Inventory of Grief (TRIG), and Perceived client sensitivity to loss scale.	
Brady, Guy, Poelstra, & Brown (1996)	Patients: No information provided<br><br>Therapists: 339 therapists (48.5% response rate), 73.7% male & 26.3% female, orientations included 45.3% eclectic, 31.7% psychodynamic, 15.1% CBT, 4.7% humanist-existential, and 2.7% other, years in practice ranged from 3 to 41.<br><br>Setting: No information provided	A two-page anonymous Psychotherapists Information Survey asking about demographics as well as information about termination was sent to participants.	Therapists reported few hindrances to successful termination aside for the hindrance of dissatisfaction with treatment results. This hindrance, the dissatisfaction of treatment, was associated with therapists' feelings of frustration, therapists' feelings of loss, future sessions given as needed, therapist decreased confidence, and therapists rescue fantasies.<br><br>Some characteristics of therapists also correlated with the hindrance of dissatisfaction of treatment results, including frequent relocation, conducting therapy to help others, practicing for self-employment, and conducting therapy to understand themselves.
Brill & Nahmani (1993)	Patients: Welfare patients receiving treatment by social work students representing the welfare population throughout Israel.<br><br>Therapists: Social work students as part of their practical training at the University of Haifa's School of Social Work. No specific demographics was provided.<br><br>Setting: No information provided.	Semi-structured interviews that were phenomenologically analyzed.	Reactions to termination: patients expressed strong reactions to termination as those experienced of attachment separations. Some reactions included bargaining, anger, grief, and acceptance. Positive feelings were expressed as well. Some subjects were matter of fact and didn't express feelings employing defense mechanisms such as repression, rationalization, projection, or denial.<br><br>Majority of the terminations were brought up anywhere from 4 weeks to 2 months in advance.<br><br>Most patients felt the termination and feelings of separation were not discussed, that what happened during treatment was not discussed, and that students tended to focus on the positive or reassure the client not to take it to heart. What was discussed was technicalities in regard to the leaving and a promise to be in contact but that workers failed to keep that promise.

			Few related that there was some emotion expressed non-verbally such as parting gifts.
Connell, Grant, & Mullin (2006)	Patients: 22,122 patients from 31 counseling services, 70% female, 87% White/Caucasian, average age of 38, reported problems included 76% anxiety and 71% depression.<br><br>Therapists: 523 therapists<br><br>Setting: Primary care counseling services.<br><br>Data drewn from CORE National Research Database for Psychological Therapies and Counseling.	Data from CORE National Research Database which included Therapist Assessment Form at intake and End of Therapy Form at termination.	50.3% unilateral ending by client before 3rd session, and those that stayed up to 6th session more likely to have a planned ending.
Cooke et al. (2020)	Patients: 205 patients tracked over 5 years, ages 18 to 70, mean age of 33.6, 68% female. They had a 4 week assessment period than 2 times a week therapy for 2 years. 133 discontinued before the 2-year mark and 40 of those provided interviews for the study; the 20 most detailed and informative ones were used. Of the 20, 11 discontinued during or after assessment phase, between session 1 to 4 and the other 9 ended before the 2-year limit.<br><br>Therapists: Patients allocated to 24 clinicians, 1 female & 7 male, 22 of them were over 40 years old. Level of education included 12 clinical psychology, 2 general psychology, 4 social work, 4 nursing, 1 psychiatry, and 1 school counselling.	Thematic analysis of interview transcripts asking about their reasons for termination.	Why terminated? For the early terminators: 5 organizational factors, 4 physical setting, 2 negative overall perception, 1 distance to clinic, 5 therapist style/technique, 5 individual characteristics, 3 therapist follow-up, 2 therapist competence, 1 lack of communication, 2 skepticism, 1 ambivalence, 3 unmet expectations, 2 practical barriers, 1 unable to recall, and 7 relationship factors.<br><br>Of the group of other terminators: 3 organizational factors, 6 physical setting, 5 therapist style/technique, 4 individual characteristics, 1 ambivalence, 5 unmet expectations, 4 practical barriers, 2 individual characteristics, and 4 relationship factors.<br><br>A few members felt termination was not discussed properly.

	Setting: Psychoanalytic community clinic.		
Corning & Malofeeva (2004)	Patients: University students and their partners.<br><br>Therapists: 24 therapists consisting of interns, practicum students, and doctoral or masters level clinicians. 87.5% were European-American, 8.3% African-American, and 4.2% Latino, with an even distribution of men and women.<br><br>File reviewed from: 739 client-therapist dyad, 60% women, mean age of 21.74 ranging from 16 to 55, 82% European American, 3.9% African-American, 3.7% Latinos, 3% Asian-Americans, 3.7% international students, 2.2% biracial, and 1.5% other.<br><br>Setting: Counseling center on campus of a private, medium-sized, midwestern university.	Forms from files were analyzed – forms consisted of demographics and concerns form, problem assessment from, and a termination form.	34.5% terminated prematurely, 31% terminated mutually, 34.5% censored cases (censored cases are those that had not terminated yet at time of the data collection or had terminated due to external circumstances, such as graduating from the university).<br><br>Premature terminations are higher at the start of treatment and decline with time. Mutual terminations are low at the start, increase through session 8, and then plateau until about session 28, after which it increases.<br><br>No correlation between individuals placed on wait-list to the type of termination, however, as sessions increase, the chance of mutual terminations increases with those that were on the wait-list (that change is lower at the start of treatment), while those that were not on the wait-list, the changes of mutual terminations are higher at the start of treatment, but decrease with additional treatment.<br><br>Previous therapy experienced decreases the changes of both mutual nd premature terminations.<br><br>An increase in canceled sessions decreased the changes of mutual terminations.<br><br>No-shows seems to predict premature terminations.<br><br>Being other referred to therapy, versus self-referred, increased both the change of premature and mutual terminations.
Craige (2002)	Patients: 121 psychoanalytic candidates who terminated treatment, 60% female and 40% male, mean age was 45 ranging from 34 to 64, length of treatment was from 1 to 16 years with a median length of 5.5 years, time since termination ranged from 1 month to 21 years with median of 2 years.	Questionnaire mailed about post-termination experience and 57 candidates consented to an additional interview (results discussed separately).	76% reported experiencing a moderate sense of loss that lasted between 6 months to a year.<br><br>Pain during termination phase highly correlated with feelings of loss afterwards.<br><br>94% reported experienced loss of the unique analytic relationship over a general sense of loss. This unique loss correlated with positive experience, successful experience, strong working alliance, intensely experienced transference, a warm relationship, sense of achieved something valuable, and moving forward in adult development.

	Therapists: Training analysts were 89% male and 11% female.<br><br>Setting: information is not provided		The intensity of painful loss correlated with length of time in analysis.<br><br>Neither sense of painful loss or loss of unique relationship correlated with significant emotional loss in childhood or adulthood.<br><br>28% reported feeling disappointed with the results of the analysis which correlated with painful loss afterwards. This high-disappointment group rated their termination as less complete and were 3x more likely to decide to terminate unilaterally.<br><br>Interview results:<br>Group A had a “good enough” analysis and mutual termination. They experienced a moderate amount of painful loss and strong loss of the unique analytic relationship. Most wished to return, others wrote letters or returned for occasional visit, and one consulted with the former analyst. They all had a positive internal image of the analyst.<br><br>Group B also had “good enough” analysis with mutual termination, although experienced difficult feelings after termination, including depressed, lost, angry, and abandoned. These painful feelings surprised them and strained their self-analytic capacities. They reentered some form of therapy/analysis.<br><br>Group C analyses ended in a stalemate, some of which ended unilaterally. Their loss experienced mixed with anger and disappointment. Majority struggled for years with this negative experience but did not label the analyst as all bad. They stated they would not return to that analyst. They also created internal images of the analyst, predominantly negative, or fluctuated.<br><br>Termination work for Group A: face and feel the loss of the analyst, understand the transferential meanings of the separation, hold onto a good enough internal image while experiencing the loss of the unique analytic relationship, take over the analysts function, and understand the loss reaction post-termination.<br>Leaving the analyst office the last time they report feelings accomplishment, pride, success, relief, liberation, and progress mixed with loss and anxiety. They successfully negotiated the work of mourning AFTER termination.

			Termination work for Group B: This group experienced a lot of challenges and difficulties post-termination, including negative transferences, unanalyzed self-object transferences, and trauma that got evoked.<br>This group did not successful mourn the analyst post termination and frequently returned to their analyst.
Cuffel et al. (2000)	Patients: 190 patients, 73.2% female, with a mean age 48.97. 50% was diagnosed with an adjustment disorder and 48.4% with depression.<br><br>Therapists & Patients: 190 patients and therapists with a mean age of 48.97.<br><br>Therapists: 50.5% doctoral-level psychologists, 46.8% social workers, and 2.6& psychiatrists.<br><br>Settings: Outpatient psychotherapy.	Survey asking both patients and therapists why they terminated treatment.	Why terminated? Patients said: 20% goals achieved, 31.6% goals partially met, 24.7% patient discontinued treatment, 3.2% provider discontinued treatment, 2.6% patient moved, 4.2% insurance changed, 3.7% no more benefits, 5.3% insurance denied further certification, and 4.7% other.<br><br>Providers said: 42% goals achieved, 20.5% goals partially met, 26.8% patient discontinued treatment, 1.1% provider discontinued treatment, 1.6% patient moved, 3.2% insurance changed, .5% no more benefits, 3.2% insurance denied further certification, and 1.1% other.<br><br>78.9% patients said their own decisions resulted in the end of treatment.<br><br>Agreement between patient-provider pairs was generally poor regarding the perceived reason for termination, except when termination was attributed to concurrent review by the managed behavioral health organization.
De Bosset & Styrsky (1986)	Patient: No information provided.<br><br>Therapists: 54 psychiatry residents in 2nd through 5th year of residency training (101 questionnaires distributed with a 54% response rate)<br><br>Setting: No information provided.	Distributed questionnaires about one of their long-term psychotherapy cases where therapy had come to an end and asked them about their experiences around length of treatment and termination.	Why terminated? 23.6%, external reasons, 11.5% goals reached, 6% patient felt benefited enough, 3.1% impasse, 1.6% drop-out.<br><br>Did therapists feel ready for patient to terminate? 29% did not agree, 55% had mixed feelings, and 16% felt patient was ready.<br><br>When applicable, termination period comprised 8% of duration of therapy.<br><br>Feelings of residents at termination: 47% work incomplete, 22% sadness, 22% warmth, 16% satisfaction with therapy.<br><br>78% left it open for patient to come back.<br><br>66% ended prior to 50th session.
DeBerry & Baskin (1989)	Patients: 228 patients (111 private patients and 117 clinic outpatients).<br><br>Therapists: 54 therapists (25 psychiatrists, 10 psychologist, 17 social workers, 2 psychiatric	450 questionaries sent asking about therapist info as well as information about the course of treatment. 12% of the	Why terminated? Excessive caseload: 24 clinic and 0 private; administrative reasons: 13 clinic and 1 private; patient referred for treatment: 6 clinic and 7 private; patient feels goals reached: 9 clinic and 39 private; therapist feels goals reached: 15 clinic and 44 private; unable to pay: 3 clinic and 7 private; other reasons: 21 clinic and 10 private (out of 216, 108 private & 108 clinic).

	nurses), mean age 42.4 and mean years post-degree experience was 11.8.<br>Orientations: 17 psychodynamic, 19 eclectic, 1 biological, 1 behavioral, 16 no orientation.<br><br>Setting: Psychiatric facilities in New York City as well as community mental health clinics.	questionnaires were returned.	Clinic patients stay longer in treatment than private patients: 33.3 months vs 22 months.<br><br>Clinic patients seen less per week at 3.5x per month vs private patients at 5.5x per month.<br><br>Clinic sessions times is less with a mean of 38 minutes vs a mean of 45.5 minutes for private patients.
Edbrooke-Childs et al. (2021)	Patients: 8,995 episodes of care, 61% female; mean age = 13.66 (SD =2.87) years, using anonymized administrative data from young people's mental health services.<br><br>Therapists: No information provided<br><br>Setting: A range of settings within the UK's mental health infrastructure, but specific details about the types of settings are not provided.	Quantitative, observational study using multilevel multinomial regression analysis controlling for age, gender, ethnicity, and referral source was conducted	Young people who meaningfully improved were less likely to have non-mutual agreement, transfer, and other reasons than case closure due to mutual agreement.
Fair & Bressler (1992)	Patients: No information provided.<br><br>Therapists: 33 doctoral students, mean age of 31.5 ranging from 24 to 41, 7 men & 26 women.<br><br>11 completed 1st year of training, 12 in their 2nd, and 10 in their 3rd.<br><br>Training sites included: 30% mental health centers, 27% private practice, 18% hospital outpatient, 12% day treatment centers, 9% college counseling centers, 9% other agencies.	Independent variable: demographics form and basic information about the patients and therapists completed by the students and a rating of the therapists performance completed by the supervisors.<br><br>Dependent variable: Termination Scale.	Termination was less planned and had greater negative emotional responses with patients who had a more severe pathological diagnosis.

	Setting: Information not provided.		
Farber, Hubbard, & Ort (2022)	Patients: 77 adult patients, 68.8% female & 28.6% male, 2.6% trans, 72.7% heterosexual, 13% bisexual, 3.9% gay, 10.4% other sexual orientation, mean age 34 ranging from 19 to 69.<br><br>Therapists: 77.1% female and 22.9% male, 75.7% White, 12.9% Black or African-American, 4.3% Asian or Asian-American, and 7.2% other.<br><br>Setting: 90.9% individual psychotherapy, 79.8% in private practice, 8.3% mental health centers, 4.2% hospital outpatient stings, 4.2% app-based services, 2.8% professional training institutes, 2.8% other settings. 52.8% seen in person and 47.2% via teletherapy.	Online Therapist Ghosting Survey with scales asking about demographics, ghosting event, feelings towards therapist, feelings about being ghosted, feelings about termination, and working alliance.	What happens when therapists ghost patients? Ghosting can be categorized as the therapist stops communicating with the patient without prior notice (no-show or doesn't return phone calls or emails.)<br><br>Findings reveal that patients make multiple attempts to contact their therapist. However, majority of them don't end up connecting with the therapist who ghosted them.<br><br>50% of patients who are ghosted wait over a year to resume therapy.<br><br>These patients assume that the ghosting either happened because they were too difficult, or because their therapist had their own challenges or life problems.<br><br>Regardless of the duration of treatment with the ghosting therapist, patients consistently express shock, frustration, anxiety, resentment, and sadness following this action. These emotions tend to diminish over time.<br><br>Ghosted patients typically view their last session with the therapist as normal, disclaim responsibility for the ghosting, and believe that the therapist should feel guilty for the ghosting event.
Fortune (1985)	Patients: "Typical clients" were young to middle-aged, middle-income adults.<br><br>Therapists: 59 MSW therapists, 35 female, 57 White, age ranged from 26 to 61 with a mean of 38, average year in practice 9.9<br><br>Setting: 25 therapists worked in private or sectarian agencies, 20 in public agencies, and 14 in solo or group private practice.	Structured in-person interview asking about length of treatment and plans for termination.	When does one use long-term or short-term treatment? For long-term treatment, more problem areas and intrapsychic rather than interpersonal or environmental problems. In contrast, environmental problems generally indicated short-term treatment.<br><br>Interpersonal issues came up as split in regard to length of treatment.<br><br>High anxiety was another indicator for long-term treatment, but many did feel that anxiety did not matter to length.<br><br>The most frequent indicator for short-term treatment was a strong support system, high motivation to change, and taking responsibility for challenges. Also indicative of short-term treatment, but less full consensus, was a strong ego by the client and if the therapist had success with previous similar cases.

			Non influencing factors to length included gender, years of experience, therapist theory, cases seen in the past year, setting of practice, client age, income, or if the treatment was voluntary.<br><br>Did they end as planned? 41% did not stick to the planned end-date, if there was one to start. Reasons they did not end as planned: incomplete goals, uncertainty of client's coping abilities, client wanting to continue, or client regressing. Consensus to end as planned was if client's goals were reached. If client felt ambivalent about ending or no progress made, came up as split in regard to extending or not.<br><br>Less than half of the sample indicated continuation decision included size of caseload, enjoying the client, and feeling tired of the client. No therapist or client characteristics impacted this decision.<br><br>When termination date was not initially set, mutual termination decisions were based one achievement of goals, client improvement, and to a lesser extent improved intrapsychic functioning. Rarely reported was lack of progress, some progress but believing that further treatment not increase the progress, or behavioral indicators of "readiness" to terminate. Other aspects that fell in the middle included client wanting to end, external circumstances, and external support availability.
Fortune A (1987)	"Typical Patient": 7 saw children, 14 adolescents, 36 young adults, 34 middle-age adults, 7 elderly, and 15 whole families.<br><br>Therapists: 59 social workers, 35 female, 57 white, age ranged from 21-61, experienced averaged 9.9 years in direct practice.<br><br>Setting: 14 worked in private practice, 19 worked in private agencies, 6 worked in sectarian agencies, and 20 in public agencies.	Students interviewed participants using a structured questionnaire asking about their termination experiences.	Therapists observed following feelings in patients: progress and success in treatment, evaluation of therapeutic experience, feelings of pride, self-accomplishment, or independence. Rarely observed was nihilistic flight, denial of termination, or regression. Patients reexperience of loss was infrequent.<br><br>Practitioners' feelings expressed included: pride in client's success and one's own therapeutic skills. Least frequent was reexperiencing of past loss, doubt about skill, disappointment in client, or relief.
Fortune, Pearlingi, & Rochelle (1992)	Patients: Patients were 86% White, 65% female, 84% over the age of 20 but ages ranged from 8 to 76 with a mean age of 34 years.	Questionnaire distributed to the therapists asking about demographics, case	Strongest client reaction reported was positive, such as pride and a sense of accomplishment. Other strong reactions were evaluation of progress and success, and evaluation of the therapy experience. The least strong reaction

	Therapists: 69 social work practitioners, 74% female, 91% White, mean age 39.4 ranging from 26 to 69.<br><br>Setting: Information not provided.	characteristics, criteria for termination, and reactions to termination.	with nihilistic flight, regression, denial of termination, expression of need for further treatment, and recreation of earlier therapeutic experiences.<br><br>Ambivalence seen in most cases. Reexperiencing of previous loss and change in the therapeutic relationship observed in majority of cases but at a very mild level.<br><br>Strongest practitioner reaction was pride or a sense of accomplishment with the client's success, pride in their own therapeutic skill, renewed sense of the therapeutic process. Sadness or a sense of loss was next in strength, but at the midpoint of the scale. Doubt, disappointment, relief, reexperiencing of past loss, and ambivalence was reported in some instances but at a mild level.<br><br>Who first raised the idea of termination was associated in patients with evaluation of progress, positive flight, change in relationship, and nihilistic flight, while associated in practitioners with sadness.
Fragkiadaki & Strauss (2011)	Patients: No information provided.<br><br>Therapists: 10 psychoanalytic and psychodynamic therapists, ranging in ages from 44 to 64, 3 males and 7 females, years in practice ranged from 6 years to 30 years.<br><br>Setting: No information provided.	Semi-structured interviews asking about termination and analyzed using grounded theory analysis.	Termination mirrored therapists' endings of their own treatment.<br><br>Therapists and patients work towards ending from the onset.<br><br>Emphasized that the development of the therapeutic relationship and the nature of the bond determines the experience of termination.<br><br>Therapists spoke of their admiration for and connection with their patients. Participants described bonding relationships where terminations had been planned and experiences of mixed feelings of intense sadness and pleasure.<br><br>Therapists expressed their desire to find out about their patients' lives after termination and tend to believe that they stay alive in their patients' minds and that work continues after the ending of therapy.<br><br>Therapists conveyed a sense of termination raising considerable intensity and range of emotion, not only for the patient but also for the therapist.<br><br>Therapists shared experiences of erratic endings with patients when the relationship had been characterized by patients' lack of commitment to the process, fluctuations, negative transference, lack of engagement, or negative outcome. Participants expressed feelings of defeat and doubt about their competence at termination of these unsettled relationships. Some discussed explicitly their sense of relief when ending a treatment that was hard to endure

			and provoked distress and anxiety in them. Participants highlighted their worry about patients leaving in an unresolved manner during these erratic terminations as well as their own sense of confusion in these lack of resolutions.<br><br>The time of termination appeared to be a crucial part of the therapy process for eight therapists in this study. Whether the therapist or the patient had initiated it, therapists spoke of a particular period when the ending is discussed and worked through. Whether a 'bonding' or 'unsettled' relationship, they spoke specifically about how they conceptualize that period of ending.<br><br>Termination was perceived as having the same impact as a personal bereavement or loss, as well as being a reminder of their own mortality.<br><br>There was a general consensus among participants that the therapy process is never finished.
Friedlander, Austin, & Cabrera (2014)	Patient: Long-term psychotherapy patients with bi-weekly or monthly sessions; no other information provided.<br><br>Therapists: 8 therapists, 7 woman and 1 man, ranging in age from 30 to 60 years, 2 identified as Latina and 7 as White. Years of experienced ranged from 5 – 25 years.<br><br>Setting: mental health clinic associated with a general hospital from 1 to 25 years. The clinic served mostly vulnerable populations.	Semi-structured focused group interview with therapists which was analyzed using Consensual Qualitative Resarch (CQR).	Who stayed in treatment indefinitely? Mainly diagnosis/level of functioning with three subcategories: SMI, personality disorders, & other chronic problems.<br><br>Contributing factors included lack of resources, poverty, and employment that is out of reach.<br><br>What were barriers to termination? Constant life crises, therapy as sole support, cultural expectations, seeing therapy as self-care, fear of leaving, attachment to patients, longstanding connections with multiple family members, and needing therapy to get their meds.
Gelman (2009)	Patients: information not provided<br><br>Therapists: 54 MSW students, mean age of 32, 87% female and 13% male, 65% White, 15% Hispanic/Latino, 12% Asian/Pacific	Questionnaire asking about termination experiences titled MSW Students' Experience with Termination.	Students felt that termination was discussed in class but less so in supervision and reported overall feeling moderately ready to undertake termination properly, majority of discussions around termination took place within two months of completing their placements.<br><br>24% experienced natural terminations. Experiences reported was that of ease and satisfaction with the work.

	Islander, 6% Black/African American, and 4% unknown.<br><br>Setting: 24% mental health, 22% school-based, 15% gerontological, 15% family and children, 9% substance abuse, 7% health care, and 7% other. Roles included 54% counseling, 26% case management, 19% psychoeducation, and 15% assessment.		35% experienced client-initiated terminations. Experiences reported were frustration, self-questioning, and a lack of closure.<br><br>93% experienced forced terminations. Most common form of termination.<br><br>In general, when termination was discussed in advanced and future plans discussed patients fared better although this type of termination evoked most feelings for both students and patients.<br><br>Students reported patients' reaction to include anxiety, depression, regression, anger, return of symptoms, or new symptoms.<br><br>Students reported that they felt guilty by forced termination. They also reported sadness, loss, anxiety about client's future.<br><br>Patients and students wanted post-termination contact but students understood the limit and the boundaries.<br><br>Students suggested having more discussion on termination and that the discussion start earlier. They also want space to express their feelings about termination.
Goldenberg (2002)	Patients: 2,889 patients, 1,175 completers and 1,714 dropout. 25% received 1 to 4 sessions, 50% received 5 to 26 sessions, 25% 27 or more sessions. Mean age 28.7, 61.1% female, 44.4% White, 34.6% diagnosed with a mood disorder and. 30.8% with adjustment disorder. 77.4% of the therapists were social workers and 12.3% were psychologists.<br><br>Therapists: No information provided.<br><br>Setting: No information provided.	Categorical regression analysis compared with linear regression analysis on aspects such as treatment outcome, medication visits, diagnosis, counselors, payer source, and type of termination.	Treatment outcome was the strongest predictor of number of sessions in the combined sample, however, when completers and drop-outs were separated, there were different correlations.<br><br>Completers ended due to improvement, and so treatment outcome lost its unique position, instead the counselor had the greatest correlation with the number of sessions, versus the other variables which were payer source, diagnosis, or medication visits.<br><br>For the dropout group, where it was mainly non-improved patients, there was no clear predictor to the number of sessions.

Gould (1977)	Patients: No information provided<br><br>Therapists: 10 student therapists who saw a total of 52 patients for 1 year with a forced termination date.<br><br>Setting: No information provided	Tape recorded face-to-face interviews asking about therapists experience of forced termination.	When did they tell patients about termination? 5 therapists brought up termination within first 3 sessions, 4 brought it up 4-7 weeks before the end, 1 told at various times.<br><br>Supervision was key component in when termination was brought up. Students felt worried about saying early in case the patient won't agree to treatment. In fact, most agreed and were relieved that there was an end date.<br><br>Student's feelings about termination: anxious, depressed, moody, guilty for learning on the patient, loss of patient or supervision, and disappointed can't complete the work. Feelings were intertwined with mixed feelings about leaving school.<br><br>Positive treatment outcome associated with client's positive affect and weaker nihilistic flight: in these cases, practitioners reported more pride and less doubt.<br><br>Difficulty in termination correlated with client's ambivalence, regression, negative affect, change in relationship, reexperiencing past loss, and expression of further need for treatment. For practitioners, sadness and reexperiencing of past loss was stronger.
Greene (1980)	Patient: No information provided.<br><br>Therapits: 92 therapists, 2/3rd was male and 2/3rd was still in training. Majority reported working from a psychoanalytic framework.<br><br>Setting: No information provided.	A battery of questionnaires about termination were mailed to 200 therapists and 92 returned them. Questionnaires including questions about demogrpahics, clinical experience, therapeutic orientatin, psychological differentiation, and the Therapist Termination Questionnaire. This Questionnaire is a 40-item self-report inventory designed to	Female clinicians expressed stronger desire to shift therapeutic relationship to a freer, less formally structured during termination. They also reported more anxiety and being more available after termination. These findings were only true for the non-analytically oriented therapists.<br><br>Analytically oriented therapists attributed primarily positive emotions to their patients during termination and tended to end with greater finality.<br><br>Trainees end therapy with greater finality than experienced clinicians.<br><br>Trainees also tend to be less satisfied with the work at completion than experienced clinicians.

		assess a variety of emotional reactions, perceptions, and techniques about termination.	
Greene & Geller (1980)	Patients: No information provided<br><br>Therapists: 71 therapists in training (including 42 psychiatry residents) and 34 experienced clinicians.<br><br>Setting: No information provided	Therapist Termination Questionnaire that addressed emotional reactions and perceptions and techniques related to termination, and Boundary-Fusion Test which assesses tendency to blur personal boundaries within the environment.	During termination, student therapists tend to minimize their own and patients' feelings related to separation. They also don't talk about possible future relapse or return to treatment.<br><br>Results also show that therapists with personal boundary difficulties tend to feel more anxiety around termination and try to get psychologically closer to their patients. However, experienced therapist did not show correlations between self-boundary management and responses to termination showing they are more able to separate between their personal and professional role.
Harai & Waehler (1999)	Participants: 143 undergraduate psychology students, 101 women and 42 men, ages ranged from 16 to 50, majority Caucasian, 53.1% freshman, 25.2% sophomores, 11.2% juniors, 10.5% seniors or 5th year, 70.6% never been in counseling and 29.4% in some counseling.	Participants listened to an audio recording of an initital session with four different conclusions and then completed the Counselor Rating From-Short (CRF-S) based on the session.	Patient's positive perception of the counselor was not shown to increase when counselors mentioned termination at the initial session.
Hartlaub, Martin, & Rhine (1986)	Patients: Of the 71 completed analyses, 34 were male and 37 were female.<br><br>Therapists: 39 analysts were sent the measure, 38 female and 1 male, 41% responded with data from 71 completed analyses, 85% of those considered successful.<br><br>Setting: No information provided.	Questionnaire sent asking about post-termination contact.	Reported that within three years of ending, 2/3 contacted them again with frequency of recontact increasing as time since termination elapsed.<br><br>Recontact included 35% brief office visit, 19% brief psychotherapy, 2% reanalysis and the other 50% made contact by letter, telephone, or other ways.<br><br>The most frequent reason given for recontact was the need to rework termination issues.<br><br>No correlation of recontact with age, diagnosis, or length of analysis.<br><br>Correlation with gender and recontact was significant with 76% of women and only 50% of men making contact again.

Hunsley, Aubry, Verstervelt, & Vito (1999)	Patients: 209 adult patients, 73% women, ages ranged from 25 – 50.<br><br>Therapists: Demographics on therapists were not provided, however, services at the clinic are provided by students from a clinical psychology program and interns from a psychology program.<br><br>Setting: University clinical psychology training clinic.	Review clinical files and telephone interviews with terminated patients. Interview asked about reasons for termination and to rate the importance of the decision to end therapy.	Why terminated? Therapists' ratings: 25.8% accomplishing goals, 20.6% no longer interested in therapy, 2.6% wanted a break from therapy, 3.1% dissatisfied with services, 11.3% referral to another service, 8.2% client moved, 5.7% no more coverage, 5.2% finished training, 13.9% where unsure why therapy was terminated.<br><br>Patients' rating: 44% accomplishing goals, 34% therapy was going nowhere, 30% therapy did not fit my idea about treatment, 30% not confident in therapist's abilities, 25% no more coverage, 25% going elsewhere, 13% no longer had time, 16% uncomfortable talking with therapists, 15% lost interest in services, and 9% therapy making things worse.<br><br>Data supports little concordance between therapists' and patients' perspectives.<br><br>Therapist was unlikely to attribute termination to client's dissatisfaction.
Hynan (1990)	Patients: 31students who initiated therpay within the first 24 weeks of the academic year. Emotional difficulties was primarily anxiety or depression.<br><br>Therapists: no data provided<br><br>Setting: Midwestern university counseling center.	Questionnaires to identify reasons for termination and experiences in therapy.	Why terminated? 4 had to study more, 1 had to work more, 2 scheduling conflicts with classes, 3 discomfort going to the clinic, 1 dislike therapist, 4 therapy not helping, 9 change in social life, 8 change in academic situation, 4 change in work life, 6 change in family life, 6 improved not due to therapy, 17 improved due to therapy, 3 difficult to talk about problems, 1 left to see other therapist, 2 discomfort after missed sessions, 3 no return after vacation.<br><br>These reasons were classified into 4 broader reasons as follows: 7/22.6% situational constraints, 12/39% discomfort with services, 27/87% life changes, and 5/16% hiatus<br><br>Late terminators were more likely to end therapy due to improvements.
Jofen-Miller & Fiori (2017)	Patients: No information provided.<br><br>Therapists: 144 licensed clinicians, average years in practice 20.7, 57% clinical psychologists, 18% social workers, 6% psychiatrists, 18% other<br><br>Orientations: 50% psychodynamically oriented, 17.5% integrative, 20% CBT, & 14% other.	Anonymous online survey consisting of 34-items related to attitudes, policies, and experience of post-termination contact.	90% of individuals (130) acknowledged experiencing some form of post-termination contact and 25% reporting (36) initiating that contact.<br><br>43.4% (62) reported having graduate training specifically addressing post-termination contact, and those had a higher rate of establishing post-termination policies that they discussed with patients. Recent graduates were more likely to report having received training with 41% having established policies for post-termination contact.<br><br>Regarding attitudes, therapists were more prone to anticipate positive rather than negative consequences of post-termination contact for both themselves and their patients. Additionally, the longer a therapist had been in practice, the

	Setting: Over 55% in private practice and additional 15% at least partly in private practice. 62.5% female, 87% white, & 73% married.		less likely they were to anticipate negative consequences of post-termination contact.<br><br>Notably, clinicians who maintained contact with their own therapists were more likely to anticipate positive consequences for both themselves and their patients.
Keleher, Oakman, Capobianco, & Mittelstaedt (2019)	Patients: 87 undergraduate students, 17 males and 69 females, average age of 20.62.<br><br>Therapists: No information provided.<br><br>Setting: Outpatient	Semi-structured interview related to termination, Basic Psychological Needs Satisfaction in Psychotherapy Scale (BNSP), Retrospective Working Alliance Inventory (RWAI-S), and termination status – planned or unplanned.	Therapy type, CBT/talk therapy etc. did not impact planned or unplanned endings.<br><br>There was no correlation shown between type of treatment provider and termination status.<br><br>Needs satisfaction was higher with the groups who had planned therapy terminations.<br><br>Working alliance was significantly higher for planned enders than unplanned enders.
Knox et al. (2011)	Patients: 12 patients, 11 woman and 1 man, all White, ages 23 to 60, 11 were trained in the mental health profession. These were all adults who had terminated from that therapy within the last three years.<br><br>Therapists: 6 female and 4 male, 8 were White, ages early 30s to late 60s, orientations included 4 psychodynamic, 2 CBT, 1 interpersonal, and 1 client-centered.<br><br>Setting: Individual outpatient psychotherapy.	Demographic forms and semi-structured interviews that were analyzed using Consensual Qualitative Research (CQR).	Five had positive terminations and 7 spoke of difficult terminations.<br><br>Positive terminations: patients generally sought therapy to address relationship concerns but also addressed stress, coping, trauma, anxiety, depression, and intrapsychic issues. They generally reported positive aspects in the relationship, with some negative aspects. They generally reported that therapy impacted them positively. Therapy generally ended for logistical reasons combined with feeling good enough to end the work. Termination was generally discussed and planned with post-termination self-care plans. During termination they both expressed emotions and reviewed the growth in therapy. They reported that termination impacted them positively and provided confidence in future coping. They also discussed grieving the loss of the therapist. They reported being open to therapy again with current therapist and understanding the importance of processing termination with their own patients.<br><br>Negative terminations: patients sought treatment for stress, coping, trauma, grief/loss, relationship concerns, anxiety, depression, and intrapsychic concerns. Therapy relationship had positive and negative qualities. Therapy impacted them positively. These terminations typically occurred because of unresolved ruptures in the relationship with logistical concerns also mentioned.

			Termination was not discussed and planned and rather a unilateral and abrupt decision made by the client. Termination typically led to a decline in the relationship. Patients reported feeling highly upset and devastated. They would go for additional therapy but only with a different therapist and some reported fear and hesitancy about future treatment.
Kramer (1986)	Patients: No information provided.<br><br>Therapists: 20 psychotherapists in long-term, open-ended individual psychotherapy in private practice (recruited using sampling technique).<br><br>Setting: Private practice settings.	Semi-structured personal interview to collect data about their views and clinical practices regarding termination and interpreted using Constant Comparative Method.	Many therapists do not have an explicitly formulated plan for concluding treatment.<br><br>Practitioners seldom brought up termination themselves.<br><br>Often practitioners didn't agree with the patient's decision to terminate.<br><br>Cues that termination is nearing exists, it's up to the therapists to look for them.<br><br>Termination should be discussed from the onset of treatment and throughout the course of treatment.<br><br>Practitioners should communicate an "open-door policy" after termination.
Ling & Stathopoulou (2020)	Patients: No information provided.<br><br>Therapists: 6 volunteer counselors recruited using purposive sampling method, 5 females and 1 male, ages ranged rom 49-66, and they volunteered between 7 months and 7 years. Orientations: 3 humanistic, 2 integrative, and 6 Adlerian.<br><br>Setting: Outpatient.	Semi-structured interviews analyzed using thematic analysis.	8 sessions for therapy is not enough to manage endings, endings experienced as challenging. Participants believed that having flexibility around number of sessions would allow positive outcomes and more manageable endings.<br><br>Counselors wanted a say in when to bring therapy to an end as opposed to it being predetermined.<br><br>All participants had to develop strategies to manage their own feelings and patients' feelings around termination.
Marmarosh, Thompon, Hill, Hollman, & Megivern (2017)	Patients: 7 males and 5 females, 4 African American, 6 Caucasiona, and 2 unknown.<br><br>Therapists: 12 therapist trainees, 6 doctoral level students and 6 master level students, 10 woman and 2 men, mean age was 29.88.	Semi-structured 60–90-minute interviews with the therapists asking about their experience with transfers and then Consensual Qualitative Research (CQR) was used for the data analysis	Transfer experience: prior therapists that are nurturing, mature, and good documentation as well as empathy, genuine, and responsibility are all facilitating personal characterizations.<br><br>Hindering characteristics include reserved, passive, poor billing practice, or not processing loss adequately.<br><br>Adaptive emotional responses that facilitate the process is patience and acceptance. Skills in managing strong and negative client affect also helped facilitate transfer.

	Setting: Two university based clinics serving the same urban population.		Feelings that hindered were insecure, anxious about rejection, concern about inexperience, and not well able to help client process loss of previous therapist.<br><br>The pathology of patients was shown to influence the transfer process, as well as client's expectations of the transfer and new therapists, namely if those expectations were met.<br><br>Clinics role – provide structure and guidance around transfer process. Some suggested requiring meeting between prior and new therapist.
Marx & Gelso (1987)	Patients: 72 patients, 81% single, 88% white, and 71% female. Average number of sessions was 10 and ranged from 2 to 51, mean age was 24.<br><br>Therapists: 16 counselors, 10 woman and 6 men, which included 5 senior staff, 2 interns, & 9 practicum students. Orientation of counselors was 25% interpersonal, 25% eclectic, and 19% psychodynamic.<br><br>Setting: University counseling center.	Termination Behavior Checklist, The Multiple Affect Adjective Check List, Importance of and Satisfaction with Termination, and Loss history assessed.	Behaviors reported during termination: looking back, looking ahead, and saying goodbye. From client's perspective, important to discuss feelings about ending together. When a history of loss is present, seems even more important to process feelings related to ending, and if omitted, can impact the entire therapy.<br><br>Patients related a lot of positive feelings regarding ending, significantly less than negative feelings.<br><br>Majority participants satisfied with how termination happened.<br><br>Findings show that greater amount of termination work done when loss was a theme, when client felt close to counselor, and when treatment was longer.
McNair, Lorr, & Callahan (1963)	Patients: 282 patients, all men under the age of 51, no central nervous system damage, less than 12 hours of therapy during the past 90 days.<br><br>If patient terminated before the 16-week mark, they were considered terminators. 176 Remainers and 106 terminators. Those who terminated for technical reasons were excluded. Within 6 weeks or less 74 individuals had quit.	Test battery to predict early termination of psychotherapy.	Remainers have a history of less impulsive and less antisocial behavior, admit more anxious behavior, are more critical of themselves, and are less likely to endorse rigid, irrational beliefs. They are also more retiring in interpersonal relationships, better educated, have better vocabularies, and therapists consider them more highly motivated for psychotherapy.<br><br>Therapists who had a higher interest in their patients' challenges had a higher rate of keeping patients in treatment – whether remainers or terminators.<br><br>Results show that female therapists, those with more experience, and therapists who had strong affections for their patients kept a higher rate of both types of patients. (The results about therapist sex appears to be specific to this sample).

	Therapists: No information provided.<br><br>Setting: Veteran Administration (VA) Hospital outpatient treatment.		
Mosher-Ashley (1994)	Patients: 298 community and nursing home patients, ages 60 to 98, 219 females and 79 males.<br><br>Therapists: No information provided.<br><br>Setting: Franklin County Mental Health Center.	Therapists' case records with quantitative analysis reported.	Who initiated termination? 59.3% patients and 31.6% therapists.<br><br>Why terminated? 10% of community residents and 8.4% nursing home cases believed client improved. Reasons community patients gave: 37.5% no need for therapy, 15% did not want therapy, 10.4% it would not help, 6.3% too painful, 4.1% moved away, 4.1% too ill, and 19% never returned to their appointment.<br><br>67.9% community residents terminated their own therapy in comparison to 26% of those in nursing home facilities
Norcross, Zimmerman, Greenberg, Swift (2017)	Patients: No information provided.<br><br>Therapists: 65 therapists, 63% male and 37% female, 76% White, 97% held a PhD and 3% were MD, number of years employed was from 17 to 52, 76% employed in university settings and 20% in independent practices. Therapists respresentative the following orientations: psychoanalytic/psychodynamic, experiential/humanistic, cognitive/cognitive-behavioral, systematic/interpersonal, multicultural, and integrative/eclectic.<br><br>Setting: University settings (76%) and independent practices (20%).	Termination Task Survey – an 80-item survey to assess termination behaviors	Strongest consensus included supporting client's progress, consolidate gains made in therapy, attribute gains to patients' effort, talking about what helped/what went well, collaborate with patient a date and pace of termination.<br><br>Strong consensus included acknowledging end of professional relationship and launching client into the future. Strong consensus on tasks including processing feelings of both participants, having client practice new skills, normalizing probability of relapse, and prompting patient to think about future without therapy.<br><br>Tasks not reaching level of consensus included post-termination behaviors of client and therapist.<br><br>Consensus on what was not done including reminding client that therapy is rarely entirely successful and using travel imagery to describe client's journey.
Olivera, Braun, Penedo, & Roussos (2013)	Patients: 17 patients consisting of 11 women and 6 men, ages 22-54, treatment ranged from 4 months to 10 years, 13 had university degrees	Snowball sampling with semi-structured face-to-face interviews analyzed using	Who initiated termination? 10 (59%) patients, 5 (29%) therapist, 2 (12%) client's couple.

	and 4 completed secondary education.<br><br>Therapists: 13 men and 4 women, 8 were psychologists, 6 psychiatrists, and 3 degrees were unknown, 12 were psychoanalysts, 2 family systems, 1 EMDR, and 2 were unknown.<br><br>Setting: Outpatient independent settings.	consentual qualitiative research (CQR).	Where they in agreement? 6 therapist and client agreed, 8 therapist agreed, 14 client agreed, 2 client did not agree, 4 therapist did not agree.<br>Why terminated? 9 change related to reasons for consultation and 8external reasons<br><br>6 (35%) therapist offered post-termination contact.
Olivera, Challu, Penedo, & Roussos (2017)	Patients: 73 patients consisting of 53 woman and 20 men within age range from 19 to 71. 43.8% had university degrees, 52.1% secondary school, 4.1% elementary school education. Occupation included: 13.7% psychotherapists, 17.8% other professionals, 31.5% clerical workers, 17.8% students, 19.2% other.<br><br>Therapists: 74% women and 26% men, 83.6% psychologists, 9.6% psychiatrists and 6.8% degrees are unknown. 41.1% were psychoanalysts, 8.2% cognitive-behavioral, 1.4% family systems, 1.4% huminstic, and 47.9% were unknown.<br><br>Setting: 84.9% in outpatient independent settings, 9.6% psychotherapy clinics, and 5.5% hospital based outpatient clinics.	Recruited via snowball sampling.<br><br>Semi-structured interviews asking about psychotherapy experience and termination, Bond Scale of the Working Alliance Inventory – Short Revised (BS-WAI-SR) and a demographics form.	Who initiated termination? 67% patients and 27% therapists.<br><br>Why terminated? 40% reached goals, 33% felt better, 11% run its course, 19% nothing to talk about, 14% needed time off, 11% therapy reached limit, 25% dissatisfied with therapy/therapist, 14% start new/different therapy, and 14% financial difficulties.<br><br>Post therapy? 56% left door open, 55% would go back to therapist, 26% would not go back to therapist.
Owen, Smith, & Rodolfa (2009)	Patients: 478 patients, 9.4% freshman, 9.2% sophomore, 12.9% juniors, 19.7% seniors, 32.9%	Expected number of sessions asked, Schwartz Outcome Scale-10 (SOS-10),	62% expected 20 or more session, 17.2% expected 11 to 20 sessions, 9.6% expected 10 or fewer, and 11.1% were not sure.

	graduate students, 1.4% other, 14.6% unknown.<br>73% female & 27% male with a median age of 22.<br>49.8% Caucasian, 14.6% Asian American, 7.3% Hispanic/Latino, 10.6% Multiethnic, .5% African-American, 1.6% Other, and 15.6% unknown.<br><br>Patients recruited during the intake process and participation was voluntary.<br><br>Therapists: No information provided.<br><br>Setting: University counseling center	College Treatment Alliance Scale (CTAS) or Individual Therapetuic Alliance Scale Revised-Short Form (ITASr-SF), Initial Emotional State and How Therapy Ended was asked.	Patients who expected 20 or more sessions reported therapy to be less effective than patients who expected 20 or less.<br><br>The anticipated number of sessions predicted the treatment outcome, even after accounting for the initial emotional state, working alliance, and the manner in which therapy concluded.<br><br>Termination status (unilateral, mutual, premature, etc.) was predicted by other clinical factors, not by the patients' anticipated number of sessions.
Pollak, Mordecai, & Gumpert (1992)	Patients: 73 patients seen over 5 years, from 1983 to 1988, 35% male, ages 18 to 49, seen weekly for about 7 months.<br><br>Therapists: 17 therapists, 11 were trainees and 6 licensed clinicians.<br><br>Setting: Outpatient mental health clinic.	At termination therapists completed a termination summary about the nature of the termination as well as a 16-item checklist adapted from Pekarik (1983).	82% remained in treatment after the 1st session and 65% continued beyond the 4th session, 45% continued after 12 sessions, 31% continued after the 24th, and 29% after the 40th session.<br><br>Why terminated? 16% improvement, 35% financial considerations, 34% dissatisfied with therapy, 26% other environmental issues, and 25% intrapsychic reasons such as fear of impact of therapy.<br><br>Therapists also reported dynamic reasons for leaving: 16% negative transference, 37% fear of dependency, 30% fear of intimacy, 19% fear of abandonment, 34% fear of loss of control, and 34% general issues of trust and safety.<br><br>Nature of termination: 73% not negotiated, 66% premature, 29% unexpected, 41% premature but anticipated, and 28% unexpected for reasons not-therapy related.
Quintana & Holahan (1992)	Patients: No information provided.<br><br>Therapists: 85 therapists, mean age 38.8 ranging from 27 to 63, 77.6% White, 60% female, 68.2%	Termination behavior checklist-therapist (TBC-T) and Counseling Outcome Questionnaire (COQ).	Termination of unsuccessful cases showed less review of the trajectory of therapy, less attempts to bring closure to the relationship, less discussion of feelings about ending. In addition, these patients devalued therapy more often.

	counseling psychology programs, and 74.1% held doctorate degrees.<br><br>Setting: 31 different university counseling centers.		There was no difference in planning for the future between successful and unsuccessful cases.<br><br>Similar to a previous finding, patients expressed more positive emotion than negative emotion when ending.
Rabu, Binder, & Haavind (2013)	Patients: 12 patients, 10 woman and 2 men, ranging from 25 to 52 years of age.<br><br>Therapists: There were 8 therapists, 5 women & 3 men, ages ranging from 49-68. Therapy ranged from 7 to 43 months and number of sessions ranged from 10 to 67.<br><br>Setting: Individual outpatient therapy.	Qualitative analysis of audio-recorded sessions, as well as analysis of post-session interviews.	Ending therapy was not straight-forward or determined by predefined criteria but through discussion together. They used structural elements such as temporary breaks to try it, tapering sessions, and possible resuming sessions when discussing termination. Therapist and patient both said they were driven by emotional reactions part of which were not discussed.<br><br>Client typically mentioned ending first, but sometimes it seemed so arranged that it was hard to decipher who actually mentioned it first. Patients tended to “hint” at it.<br><br>In general, ended was broached with a lot of “we” in it.<br><br>Working alliance tends to be at stake toward the end of treatment.<br><br>Maintaining working alliance is important through the end so that gains remain and that patients retain positive memory of the therapist.<br><br>Ending seems to be less about looking back to assess what happened and more about the future without therapy. Instead of discussing the work, they celebrate the relationship and doing it together.
Rabu & Haavind (2012)	Patient: 35-year-old female<br><br>Therapist: 54-year-old male who worked in a public outpatient clinic<br><br>The patient/therapist dyad was taken from existing data that consisted of 18 therapists and 40 patients. This case was selected strategically because the therapist wanted to end treatment when the patient didn’t feel ready.<br><br>Setting: Outpatient setting.	Alliance was measured with the Working Alliance Inventory (WAI), outcome measured with Outcome Questionnare 45 (OQ-45), both had post-therapy interviews, all sessions were audio recorded and used in the analysis.	When is appropriate to end when there is no set date from the onset? Relational quality evokes fear in ending.<br><br>The discrepancy between therapist and client thoughts about when to end was not addressed but rather postponed and revisited again later.<br><br>Structural elements like preparations for a break for vacations and reducing the frequency of sessions were used to test experiential qualities, such as how the client managed life without therapy.<br><br>Therapists and patients both put emphasis on maintaining a ‘good enough’ bond through the termination process. Patient’s autonomy displayed improvement and by accepting the ending, the patient saw and acknowledged their progress throughout treatment.

			Maintain alliance throughout that process.
Rabu & Haavind (2018)	Patients: 37 patients, 24 woman and 13 men, ages ranging from 25 to 62, therapy ranged from 5 to 62 months, naturalistic clinical sample.<br><br>Therapists: Therapists were 14 psychologists and 2 psychiatrists, mean of 30 years experience, 12 women and 4 men, ages 49 to 68. Orientations included: psychodynamic, behavioral, cognitive, humanistic, systemic, or other.<br><br>Setting: Psychotherapy in naturalistic settings – public-funded private practices or public outpatient clinics.	Patients interviewed in both short and long-term treatment, specifically when ending was not decided from onset.<br><br>Thematic case-by-case analyses of their narratives.	Most patients felt therapy was productive and that there was mutual engagement. Coming to terms with challenges in life and therapy made patients ready to end. Some patients had unresolved issues such as wondering if another therapist could have helped more (most felt therapist was a good match), feeling pushed away by the therapist (most felt heart about their feelings regarding ending), having to decide themselves about termination, feeling unworthy as a client and therefore not sure when to end, or wanting to end without therapists' approval. These unresolved issues were put away during termination. Making meaning of the ending of therapy was personal and emotional and not always shared with the therapist.<br><br>Patients felt they needed time to come to terms with the separation. Others said it was good to end quickly once they realized they were finished. Some continued to use their internal therapist after termination. Some saw ending as relief and an affirmation that they had improved.<br><br>Majority felt it was their responsibility to bring up ending.
Rabu, Haavind, & Binder (2013)	Patients: 10 women & 2 men ranging from ages 25 to 52.<br><br>Therapists: 5 woman and 3 men ranging in age from 4 to 68, mean years of practice was 30, 7 were psychologists and 1 was a psychiatrist.<br><br>12 therapist / client dyads where ending was not predetermined.<br><br>Therapy ranged from 7 to 43 months.<br><br>Setting: Outpatient setting	Audio recording from sessions and post-therapy interviews with patients and therapists. Analyzed using hermeneutical-phenomenological approach.	Lots of metaphors seem to be used regarding the end. These metaphors centered around accomplishments reached, optimism for life after therapy, and reality of new challenges arising. Ending shows discussion of growth in affective and relational aspects.
Renk & Dinger (2002)	Patients: 366 client records, 31.9% men and 68.1% women, mean age 31.92 ranging from 17 to 74, 84% White.	Patients' files reviewed including reasons for termination, Beck Depression Inventory	Why terminated? 35.8% gave no notice or reason, 23.5% mutual agreement due to satisfaction, 19.9% external circumstances, 11.5% found treatment elsewhere, 8.5% dissatisfied, .8% therapist terminated.

	Therapists: Graduate student therapists.<br><br>Setting: University-based clnic	(BDI), and number of sessions attended.	Patients who ended for no reason or notice and those that found treatment elsewhere endorsed higher levels of depression on the BDI.<br><br>Patients whose therapist terminated therapy attended the most sessions than all other groups.<br><br>Patients who had mutual termination based on success had more therapy sessions than those that stopped unilaterally, found therapy elsewhere, and those that external difficulties which caused them to terminate.
Roe David (2007)	Patients: 82 patients who completed at least 6 months in psychodynamic private practice therapy, 79% female, average number of months in therapy 27.70, and average months since therapy ended 17.93.<br><br>Therapists: No information provided<br><br>Setting: Not specified	Convenience Sampling.<br><br>Scale measuring: Timing to termination, Reasons for terminating therapy (RTTS), Feelings towards terminating therapy (FTTS), and Satisfaction with Treatment.	When did treatment end? 40% treatment ended on time, 37% treatment ended early, 23% treatment ended late.<br><br>Length of treatment longer with those who said it lasted too long.<br><br>Those that ended early or late both reported less satisfaction.<br><br>Reasons for those that ended too early included financial, mismatch, therapist lack of containment, external reasons, and lack of faith in treatment.<br><br>Reasons for those that were too long included discomfort with therapist, hope treatment will improve, dependence on therapist, nature of treatment determining its length, and client taking their time.<br><br>56% reported that therapist opposed their decision to end and 44% therapist expressed some degree of acceptance.
Roe, Dekel, Harel, & Fennig (2006a)	Patients: 84 patients, 79% female, 36% ranged in age from 20 -30, 54% from 30-40, and 10% older than 40, 46% married, 48% single, and 6% divorced, mean number of months in therapy being 27.7<br><br>Therapists: No information provided.<br><br>Setting: Outpatient private practice	Convenient sampling, Reasons for Terminating Therapy Scale (RTTS), open-ended questions asking why therapy ended, Satisfaction with Treatment Scale, and Termination Initiation and Length of Termination Process.	Why terminated treatment? 54.6% circumstantial including financial and external, 45.5% achieved goals, 36.4% dissatisfaction with therapist, 29.9% dissatisfaction with therapy, 14.3% need for independence, 11.7% busy with new relationship.<br><br>Quantitative results revealed that: (1) patients initiated the termination in a little over two-thirds of the cases; (2) the most frequent reasons for termination were the accomplishment of goals, circumstantial constraints and dissatisfaction; and (3) client satisfaction was positively related to positive reasons for termination such as improvement, and negatively related to negative reasons for termination such as dissatisfaction with psychotherapy and the therapist.
Roe, Dekel, Haral, Fennig, Fennig (2006b)	Patients: 84 patients, 79% female, 36% ranged in age from 20 -30, 54% from 30-40, and 10% older	Convenient sampling, Feelings toward Termination Scale	44% positive feelings at termination when it was a practice of independence, 42.5% reflecting positive aspects of the relationship, 35.3% reflected positive

	than 40, 46% married, 48% single, and 6% divorced, mean number of months in therapy being 27.7<br><br>Therapists: No information provided.<br><br>Setting: Outpatient private practice	(FTTS), Satisfaction with Treatment Scale, Initiation and Duration of Termination, and Semi-structured questions asking about feelings about termination. The data was analyzed using open-coding case analysis, axial coding, and creating a synthesis.	gains made in therapy, and 7.4% circumstantial relief such as financial benefits.<br><br>38.1% negative feelings related to loss of meaningful relationship, 29.6% relationship difficulties, 28.4% premature termination and need for further treatment, and 19.7% disappointment, dissatisfaction, and feelings of failure such as therapist not accepting/respecting their decision to end.<br><br>14.7% mentioned therapist leaving the door open.<br><br>11.8% mentioned processing termination.
Safarzade, Boogar, Talepasand (2015)	Patients: 317 individuals recruited by convenient sampling, 46.1% female and 53.9% male. Mean age was 32.91 ranging from 18 to 59.<br><br>Therapists: 27.8% had a master of psychology, 14.8% master of counseling and guidance, 36.9% doctor of psychology, 6.6% doctor of counseling, and 13.9% psychiatrist. (No other therapists' info provided).<br><br>Setting: Psychiatry and psychology clinics	Demographic questionnaires created by researchers and the Outcome Questionnaire (OQ)	The findings show that successful termination is influenced by factors such as gender, age, level of education, income status, referral source, access to treatment, therapist's level of education, type of psychotherapy, and the patient's perceived severity of the problem. The cost of treatment and clinical diagnosis did not emerge as predictors of successful termination of psychotherapy.
Schen, Raymond, & Notman (2013)	Patients: Patients had a full range of Axis I and II diagnoses.<br>The GAF (Global Assessment of Function) ranged from a low of 31 to a high of 70, with the majority of patients falling in the 50s–60s.<br><br>Therapists: 23 (of 25) psychiatry residents voluntarily responded to the survey distributed.	Online questionnaire with free response questions about their transfer experiences.	Transfer terminations: Residents encounter challenges in communicating their departure to patients and frequently postpone this task. Given the development of closeness and attachment in long-term therapeutic relationships, residents often express a mix of emotions, including guilt, uncertainty, anxiety, sadness, feelings of loss, and occasional relief as they prepare patients for transfer. Outgoing residents may experience anxiety when acknowledging and addressing both their patients' positive feelings and their own emotions. Conversely, incoming residents may feel uneasy being compared to the previous therapist and might confront the patient's negative emotions during the transfer, as well as the loss of the prior therapeutic connection.

	Setting: No information provided.		It is recommended to incorporate teaching on the dual aspects of transferring care to enhance understanding and response to this transition for both patients and therapists. This educational effort should encompass addressing the associated stresses and providing recommendations for effective management.<br><br>More anxiety is reported when they don't know who the new therapist will be and they can't arrange a person-to-person handoff.
Schulman & Kay (1989)	Patients: No information provided.<br><br>Therapists: 14 former residents and 16 former residents' supervisors were sent the survey although 9 former residents and 16 former residents' supervisors responded.<br><br>Setting: Transfer from residents' training sites to their private practices.	Questionnaires sent to psychiatry residents and supervisors who worked with residents to ask about attitudes and clinical expereinces transferring patients to private practices.	Transfers: Most supervisors agreed that it was beneficial for residence to transfer their patients to their practice as a continuity of care, while some supervisors thought it was a transference/countertransference issue and not beneficial.<br><br>Some cases the patient initiated the transfer while in most cases it was the resident's idea.<br><br>In most cases the resident discussed it at length with their supervisors.
Shafran et al. (2020)	Patients; 23 pateints who initiated termination, had at least 8 sessions, and attended the termination session. Of those,10 identified as satisfied and 5 as unsatisfied. The satisfied consisted of 6 females and 4 males ranging in ages from 22-45 and the unsatisfied consisted of 1 female & 4 males ranging in ages from 18-45.<br><br>Therapists: 12 counseling psychology doctoral students with at least 2 years in practica, 7 female and 5 male ranging in ages from 25-34.<br><br>Setting: Psychology department clinic	Termination Behavior Checklist (TBC), Outcome Questionnaire 45.2 (OQ), Inventory of Interpersonal Problem – 32 (IIP), and Post-termination interviews.	In satisfied cases, there was more evidence of behaviors related to termination. With these cases therapists and patients mutually looked back together to review therapy and describe changes that occurred, therapists addressed unresolved issues, dyads discussed the relationship, discussed possible upcoming challenges after therapy, discussed future therapy plans and an open goodbye was said.<br><br>In unsatisfied cases, final sessions were similar to those in satisfied cases, however, in contrast patients avoided discussing termination and devalued therapist or therapy. They also did not want to engage in any looking forward behaviors. They both didn't discuss many feelings about termination and no nonverbal gesture of gratitude was shown.
Sullivan, Zeff, & Zweig (2018)	Patients: Older adults.	Online survey asking about demographics and the Psychotherapy	Which termination guideline was important when working with depressed older adults? Reviewing gains made in treatment, terminating therapy when the patient reached their goals, and providing advance notice and discussing

	Therapists: 96 licensed psychologists who provided services to older adults. 93% where White, 67% female, 83% held a PhD and 14% a PsyD, 59% geropsychologists. Mean age was 52 ranging from 26-84. Modalities included 42% CBT, 17% Psychodynamic, 16% Eclectic, 14% Integrative, 3% Behavioral, 5% Interpersonal, 1% Humanistic, and 1% Family Systems.<br><br>Setting: Outpatient setting.	Termination Practices Rating Scale (PTPRS).	the end together. Psychologists rated specific approaches to ending treatment (discussing termination with family & clarifying post-termination relationship/no open-door policy) as significantly more appropriate when working with an older adult with personality disorder versus an older adult without personality disorder.<br><br>Clinician factors: theoretical orientation – those that were integrative were less likely to endorse a specific set of termination practices as appropriate. Those that identified as geropsychologists were more likely to endorse protective approaches to termination.
Todd, Deane, & Bragdon (2003)	Patients: 123 patients, mean age at termination was 26.56, 2/3rd were women.<br><br>Therapists: 63 therapists, mean age at termination was 29.07, therapists ranged from doctoral students in the first through the fifth year of training.<br><br>Setting: Clinical psychology training clinic at a university.	Forms in the clinic's database, Therapist Case Summary (TCS), Client Termination Form (CTF), Global Severity Index (GSI), and Development of the Reasons for Termination Coding System (RTCS).	Why terminated?<br>Improvement: Cl and Th: 12, Cl only: 2, Th only: 15, Neither: 71<br>Cl negative: Cl and Th: 2, Cl only: 6, Th only: 2, Neither: 90<br>Cl environment: Cl and Th: 41, Cl only: 12, Th only: 3, Neither: 44<br>Th environment: Cl and Th: 19, Cl only: 4, Th only: 9, Neither: 68<br>Other: Cl and Th: 3, Cl only: 15, Th only: 7, Neither: 75<br><br>Therapists more likely than patients to endorse "improvement" and patients more likely to endorse "client environmental" or "other" reason.<br><br>Basically, greater concordance than Hunsley et al. (1999) study, here, with most reasons given the dyads were in agreement besides what delineated above.
Tryon & Kane (1993)	Patients: 103 college student patients, 65 women & 38 men who took part in at least 10 sessions.<br><br>Therapists: 4 PhD psychologists & 6 practicum trainees.<br>The psychologists: 100% were White, mean age 43.75, 100% female, 2 psychodynamic and 1 client centered.<br>The trainees: 2 men and 4 women, mean age 33.33, 100% psychodynamic.	Working Alliance Inventory (WAI-S) and status of termination determined by records.	Unilateral terminators had fewer sessions than mutual terminators.<br><br>Therapists' ratings of the WAI were lower for those that later terminated unilaterally.<br><br>Therapists' ratings were lower for both groups in comparison to patients' ratings.<br><br>Patients' WAI ratings did not differ by the type of termination.

	Setting: University counseling cetner.		
Tryon & Kane (1995)	Patients: 109 college students, 81 women and 25 men, 90 students, 83% Caucasian, 11% Hispanic, 3% Asian, 3% African-American. Average age was 22.2 years.<br><br>Therapists: 10 therapists including 4 females with doctorates, 1 had a counseling doctorate and 3 were clinical psychologists. The rest were trainees – 2 from clinical psychology and 4 counseling psychology programs. Of the doctorates, 3 were psychodynamic oriented and 1 was client centered.<br><br>Setting: Counseling center	Likert scale to assess involvement in session and relatedness, the Working Alliance Inventory (WAI-S), and attendance was assessed by using records.	Patients who relate well at intake more likely rated for stronger alliance.<br><br>Patient's involvement in therapy impacts outcome.<br><br>Termination is more likely to be unilateral when therapeutic alliance is weaker.<br><br>Patients who would terminate unilaterally rated their alliances more negatively than did those who would later terminate mutually. Therapists rated alliances with patients who would later terminate unilaterally more negatively than with patients who would later terminate mutually.
Vaslamatzis, Markidis, & Katsouyanni (1989)	Patients: 27 patients for 30 session protocol, 18% dropped out between the 21st and the 28th session when termination was already brought up, 30% continued beyond the protocol, and 52% completed treatment.<br><br>Therapists: No information provided.<br><br>Setting: Psychoanalytic psychotherapy unit of the Athens University Department of Psychiatry.	Supervisors rated therapists on factors which were divided into detached therapist, self-confident therapist, and guilt-prone therapist. Factor analyses were done with these ratings. Patients received a suitability score during the first 5 sessions based on Davanloo (1980). This measured their suitability for brief psychotherapy.	Patients who terminated before the 30th sessions, tend to have therapists rated as non-self-confident.<br><br>Completes had significantly higher suitability score compared to continues, which had higher than late dropouts. The more a patient was suitable for brief psychotherapy, the more likely the course of treatment will be completed and will be enough to help the individual.
Weil, Katz, & Hilsenroth (2017)	Patients: 30 outpatient adults at a university-based community clinic,	Vanderbilt Psychotherapy Process	Therapists and patients view of patient participation in a termination session related to patient ratings of good and productive termination sessions.

	23 female and 7 male, 18 single, 8 married, and 4 separated, mean age 28.7, majority (53.3%) were diagnosed with a mood disorder.<br><br>Therapists: 27 doctoral students comprising of 13 males & 14 females, between the middle 20s to middle 30s in age, and they were all under a minimum of 3.5 hours of supervision per week.<br><br>Setting: University based community clinic.	Scale-Short Form (VPPS-S), Session Evaluations Questionnaire (SEQ), Brief Symptom Inventory (BSI), Schwartz Outcome Scale (SOS), Social Adjustment Scale (SAS).	Meaning, in successful treatments, patients were more engaged in the termination process.
Westmacott & Hunsley (2010)	Patients: 973, 34% men and 66% women.<br><br>Therapists: 275 (28.3%) individuals terminated with a social worker, counselor, or psychotherapist, 276 (28.4%) terminated with a psychologist, 239 (24.6%) terminated with a general practitioner, and 183 (18.8%) terminated with a psychiatrist.<br><br>Setting: Various mental health settings across Canada.	Interviews about reasons for termination and demographic variables.	Why terminated? 43% felt better, 13% completed recommended therapy, 14% therapy not helpful, 7% problem will get better without more help, 5% financial constraints, .4% embarrassed to see therapist, 5.1% didn't want therapy, 2.1% external constraints, 4.2% service no longer available, 7.2% not comfortable with therapist approach, and 19.3% other reasons.
Wesmacott & Hunsely (2017)	Patients: No information provided.<br><br>Therapists: 269 psychologists, 80.7% doctoral degree, 13% master's degree, 6.3% unknown. 58% female, 34.9% male, & 7.1% unknown.89.6% independent practice, 4.1% supervised practice, 6.3% unknown. Mean age was 49.8 with a range from 25 to 71.<br><br>Setting: No information provided.	Online survey asking about reasons for termination, type of termination, feelings related to termination, and strategies for retention of patients.	13% unilaterally terminated before the third therapy session and 20% after the third session. 66.7% mutual terminators.<br><br>3.4% of psychologist reported that nobody terminated before the 3rd session.<br><br>42.3% of psychologists reported that 5% or less terminated before the 3rd session.<br><br>3.4% of psychologists reported that no client unilaterally terminated after the 3rd session<br><br>22% reported that 5% or less terminated unilaterally after the 3rd session.

			6.8% of psychologists reported that less than 20% terminated mutually, however, 40.2% of psychologist reported that 80% or more of their patients terminated mutually.<br><br>Why did patients unilaterally terminate? For those within the first 3 sessions, biggest reason was lack of motivation. For those who unilaterally terminated after the 3rd session, reason given was goal reached or symptoms improved. Almost all psychologist reported that they focus on building an alliance as a retention strategy.
Westmacott, Hunsley, Best, Rumstein-McKean, Schindler (2010)	Patients: 83 patients of whichs 19 males and 64 females with a mean age of 31.7, majority were White and majority were highly educated, 28.9% were students, 37.3% were employed full-time, 20.5% were unemployed, 12% employed part-time, and 1.2% homemakers.<br><br>Therapists: 35 therapists, 28 women and 7 men. Therapists were students and interns in a doctoral program who provided therapy to between one and 11 patients.<br><br>Dyads: 31 dyads agreed that termination was unilateral by the client, 52 agreed it was mutual, 24 did not agree on the type of termination and therefore their data was not used.<br><br>Setting: University clinical psychology training clinic.	Demographic data, Symptom Checklist-10 (SCL-10), Global Assessment of Functioning Scale (GAF), Working Alliance Inventory (WAI-S), Barriers to Treatment Participation Scale (BTPS), & Reasons for termination.	Unilateral terminators attended significantly fewer sessions.<br><br>Unilateral terminators reported a significant decline in distress although mutual terminators reported a similar decline. Posttherapy mutual terminators were significantly less distressed.<br><br>On the GAF, therapists reported improvement for the mutual terminators and no improvement for the unilateral terminators.<br><br>Posttherapy, mutual terminators were rated by therapists as having higher functioning.<br><br>In mutual terminations, the dyads agreed on reasons for termination, but in unilateral terminations client's rated "therapy was going nowhere" higher than therapists rated it.<br><br>In mutual termination the WAI was higher rated by the dyad, with these ratings showing more discrepancy under unilateral terminations.<br><br>In general, patients tended to rate the working alliance higher than the therapists.<br><br>Unilateral terminators reported more barriers to treatment than mutual terminators and overall patients reported more barriers in treatment than therapists.

Note: Tx or tx = treatment, OQ = Outcome Questionnaire, COQ = Counseling Outcome Questionnaire, WAI = Working Alliance Inventory, WAI-S = Working Alliance Inventory Short Version, CQR = Consensual Qualitative Research, TBC-T = Termination behavior checklist-therapist, COQ = Counseling Outcome Questionnaire, BDI = Beck Depression Inventory, GSI = Global Severity Index, RTTS = Reasons for Terminating Therapy Scale, TBC = Termination Behavior Checklist, TBC-T – Termination Behavior Checklist – Therapist, TCS – Therapist Case Summary, GAF = Global Assessment of Functioning Scale, SEQ = Session Evaluations Questionnaire, SCL-10 = Symptom Checklist-10, SAS = Social Adjustment Scale, SOS = Schwartz Outcome Scale, BSI = Brief Symptom Inventory, TTQ = Therapist Termination Questionnaire, RTCS = Development of the Reasons for Termination Coding System, CTF = Client Termination Form, BTPS = Barriers to Treatment Participation Scale, TRIG = Texas Revised Inventory of Grief, FTTS = Feelings towards terminating therapy, IIP = Inventory of Interpersonal Problem – 32, VPPS – S = Vanderbilt Psychotherapy Process Scale-Short Form, PTPRS = Psychotherapy Termination Practices Rating Scale, CRF – S = Counselor Rating From-Short.

References

Baum, N. (2005). Correlates of Clients' emotional and Behavioral Responses to Treatment Termination. *Clinical Social Work Journal*, *33*(3), 309–326. https://doi.org/10.1007/s10615-005-4946-5

Baum, N. (2006). End-of-Year Treatment Termination: Responses of Social Work Student Trainees. *British Journal of Social Work*, *36*(4), 639–656. https://doi.org/10.1093/bjsw/bch253

Baum, N. (2007). Therapists' Responses to Treatment Termination: An Inquiry into the Variables that Contribute to Therapists' Experiences. *Clinical Social Work Journal*, *35*(2), 97–106. https://doi.org/10.1007/s10615-006-0066-0

Bhatia, A., & Gelso, C. J. (2017). The termination phase: Therapists' perspective on the therapeutic relationship and outcome. *Psychotherapy*, *54*(1), 76–87. https://doi.org/10.1037/pst0000100

Boyer, S. P., & Hoffman, M. A. (1993). Counselor affective reactions to termination: Impact of counselor loss history and perceived client sensitivity to loss. *Journal of Counseling Psychology*, *40*(3), 271–277. https://doi.org/10.1037/0022-0167.40.3.271

Brady, J. L., Guy, J. D., Poelstra, P. L., & Brown, C. K. (1996). Difficult Good-Byes: *Psychotherapy in Private Practice*, *14*(4), 65–76. https://doi.org/10.1300/j294v14n04_05

Brill, M. B., & Nahmani, N. N. (1993). Client' Responses to Separation from Social Work Trainees. *Journal of Teaching in Social Work*, *7*(2), 97–111. https://doi.org/10.1300/j067v07n02_08

Connell, J., Grant, S., & Mullin, T. (2006). Client initiated termination of therapy at NHS primary care counselling services. *Counselling and Psychotherapy Research*, *6*(1), 60–67. https://doi.org/10.1080/14733140600581507

Cooke, J., Ivey, G., Godfrey, C., Grady, J. N., Dean, S., Beaufoy, J., & Tonge, B. J. (2020). Patient-reported reasons for discontinuing psychotherapy in a low-cost psychoanalytic

community clinic. *Counselling and Psychotherapy Research*, *21*(3), 697–709. https://doi.org/10.1002/capr.12352

Corning, A. F., & Malofeeva, E. V. (2004). The application of survival analysis to the study of psychotherapy termination. *Journal of Counseling Psychology*, *51*(3), 354–367. https://doi.org/10.1037/0022-0167.51.3.354

Craige, H. (2002). Mourning Analysis: The Post-Termination Phase. *Journal of the American Psychoanalytic Association*, *50*(2), 507–550. https://doi.org/10.1177/00030651020500021001

Cuffel, B. J., McCulloch, J., Wade, R., Tam, L., Brown-Mitchell, R., & Goldman, W. M. (2000). Patients' and providers' perceptions of outpatient treatment termination in a managed behavioral health organization. *Psychiatric Services*, *51*(4), 469–473. https://doi.org/10.1176/appi.ps.51.4.469

De Bosset, F., & Styrsky, E. (1986). Termination in Individual Psychotherapy: A survey of Residents' experience. *The Canadian Journal of Psychiatry*, *31*(7), 636–642. https://doi.org/10.1177/070674378603100707

DeBerry, S., & Baskin, D. S. (1989). Termination Criteria in Psychotherapy: A comparison of private and public practice. *American Journal of Psychotherapy*, *43*(1), 43–53. https://doi.org/10.1176/appi.psychotherapy.1989.43.1.43

Edbrooke-Childs, J., Da Silva, L. C., Čuš, A., Liverpool, S., Mota, C. P., Pietrabissa, G., Bardsley, T., Sales, C., Ulberg, R., Jacob, J., & Ferreira, N. (2021). Young people who meaningfully improve are more likely to mutually agree to end treatment. *Frontiers in Psychology*, *12*. https://doi.org/10.3389/fpsyg.2021.641770

Fair, S. M., & Bressler, J. M. (1992). Therapists-Initiated termination of psychotherapy. *The Clinical Supervisor*, *10*(1), 171–189. https://doi.org/10.1300/j001v10n01_14

Farber, B. A., Hubbard, E., & Ort, D. (2022). Patients' experiences of being "ghosted" by their psychotherapists. *Psychotherapy*, *59*(4), 545–553. https://doi.org/10.1037/pst0000454

Fortune, A. E. (1985). Planning duration and termination of treatment. *Social Service Review*, *59*(4), 647–661. https://doi.org/10.1086/644337

Fortune, A. E. (1987). Grief only? Client and social worker reactions to termination. *Clinical Social Work Journal*, *15*(2), 159–171. https://doi.org/10.1007/bf00752909

Fortune, A., Pearling, B., & Rochelle, C. (1992). Reactions to termination of individual treatment. *Social Work*, *37*(2), 171–178. https://doi.org/10.1093/sw/37.2.171

Fragkiadaki, E., & Strauss, S. M. (2011). Termination of psychotherapy: The journey of 10 psychoanalytic and psychodynamic therapists. *Psychology and Psychotherapy: Theory, Research and Practice*, *85*(3), 335–350. https://doi.org/10.1111/j.2044-8341.2011.02035.x

Friedlander, M. L., Austin, C. L., & Cabrera, P. (2014). When psychotherapy is indefinite and there is no final outcome: Case study of a community mental health clinic. *Psychotherapy*, *51*(4), 580–594. https://doi.org/10.1037/a0036060

Gelman, C. R. (2009). MSW Students' Experience with Termination: Implications and Suggestions for Classroom and Field Instruction. *Journal of Teaching in Social Work*, *29*(2), 169–187. https://doi.org/10.1080/08841230802238328

Goldenberg, V. (2002). Ranking the correlates of psychotherapy duration. *Administration and Policy in Mental Health and Mental Health Services Research*, *29*(3), 201–214. https://doi.org/10.1023/a:1015187324925

Gould, R. P. (1977). Students' experience with the termination phase of individual treatment∗. *Smith College Studies in Social Work*, *48*(3), 235–269. https://doi.org/10.1080/00377317809516515

Greene, L. R. (1980). On Terminating Psychotherapy: More evidence of Sex-Role related Countertransference. *Psychology of Women Quarterly*, *4*(4), 548–557. https://doi.org/10.1111/j.1471-6402.1980.tb00725.x

Greene, L. R., & Geller, J. D. (1980). Effects of therapists' clinical experience and personal boundaries on termination of psychotherapy. *Academic Psychiatry*, *4*(1), 31–35. https://doi.org/10.1007/bf03399750

Harari, M. J., & Waehler, C. A. (1999). The effect of first session attention to termination on counselor social Influence rating. *Journal of College Student Psychotherapy*, *13*(4), 67–79. https://doi.org/10.1300/j035v13n04_06

Hartlaub, G. H., Martin, G. C., & Rhine, M. W. (1986). Recontact with the Analyst Following Termination: A Survey of Seventy-One Cases. *Journal of the American Psychoanalytic Association*, *34*(4), 895–910. https://doi.org/10.1177/000306518603400406

Hunsley, J., Aubry, T., Verstervelt, C. M., & Vito, D. (1999). Comparing therapist and client perspectives on reasons for psychotherapy termination. *Psychotherapy*, *36*(4), 380–388. https://doi.org/10.1037/h0087802

Hynan, D. J. (1990). Client reasons and experiences in treatment that influence termination of psychotherapy. *Journal of Clinical Psychology*, *46*(6), 891–895. https://doi.org/10.1002/1097-4679(199011)46:6

Jofen-Miller, S., & Fiori, K. L. (2017). The impact of psychotherapist training and experience on posttermination contact. *Psychotherapy*, *54*(1), 114–122. https://doi.org/10.1037/pst0000105

Keleher, B., Oakman, J. M., Capobianco, K., & Mittelstaedt, W. H. (2017). Basic psychological needs satisfaction, working alliance, and early termination in psychotherapy. *Counselling Psychology Quarterly*, *32*(1), 64–80. https://doi.org/10.1080/09515070.2017.1367271

Knox, S., Adrians, N., Everson, E., Hess, S. A., Hill, C. E., & Crook-Lyon, R. E. (2011). Clients' perspectives on therapy termination. *Psychotherapy Research*, *21*(2), 154–167. https://doi.org/10.1080/10503307.2010.534509

Kramer, S. (1986). The termination process in open-ended psychotherapy: Guidelines for clinical practice. *Psychotherapy*, *23*(4), 526–531. https://doi.org/10.1037/h0085652

Ling, L., & Stathopoulou, H. (2020). An exploration of ending psychotherapy: The experiences of volunteer counsellors. *Counselling and Psychotherapy Research*, *21*(3), 729–738. https://doi.org/10.1002/capr.12379

Marmarosh, C. L., Thompson, B. J., Hill, C. E., Hollman, S., & Megivern, M. (2017). Therapists-in-training experiences of working with transfer clients: One relationship terminates and another begins. *Psychotherapy*, *54*(1), 102–113. https://doi.org/10.1037/pst0000095

Marx, J. A., & Gelso, C. J. (1987). Termination of individual counseling in a university counseling center. *Journal of Counseling Psychology*, *34*(1), 3–9. https://doi.org/10.1037/0022-0167.34.1.3

McNair, D. M., Lorr, M., & Callahan, D. M. (1963). Patient and therapist influences on quitting psychotherapy. *Journal of Consulting Psychology*, *27*(1), 10–17. https://doi.org/10.1037/h0039857

Mosher-Ashley, P. M. (1994). Therapy termination and persistence patterns of elderly clients in a community mental health center. *The Gerontologist*, *34*(2), 180–189. https://doi.org/10.1093/geront/34.2.180

Norcross, J. C., Zimmerman, B. E., Greenberg, R. P., & Swift, J. K. (2017). Do all therapists do that when saying goodbye? A study of commonalities in termination behaviors. *Psychotherapy*, *54*(1), 66–75. https://doi.org/10.1037/pst0000097

Olivera, J., Braun, M., Penedo, J. M. G., & Roussos, A. (2013). A qualitative investigation of former clients' perception of change, reasons for consultation, therapeutic relationship, and termination. *Psychotherapy*, *50*(4), 505–516. https://doi.org/10.1037/a0033359

Olivera, J., Challú, L., Penedo, J. M. G., & Roussos, A. (2017). Client–therapist agreement in the termination process and its association with therapeutic relationship. *Psychotherapy*, *54*(1), 88–101. https://doi.org/10.1037/pst0000099

Owen, J., Smith, A., & Rodolfa, E. (2009). Clients' expected number of counseling sessions, treatment effectiveness, and termination status: using empirical evidence to inform

session limit policies. *Journal of College Student Psychotherapy*, *23*(2), 118–134. https://doi.org/10.1080/87568220902743660

Pollak, J., Mordecai, E. M., & Gumpert, P. (1992). Discontinuation from Long-Term Individual Psychodynamic Psychotherapy. *Psychotherapy Research*, *2*(3), 224–234. https://doi.org/10.1080/10503309212331332994

Quintana, S. M., & Holahan, W. L. (1992). Termination in short-term counseling: Comparison of successful and unsuccessful cases. *Journal of Counseling Psychology*, *39*(3), 299–305. https://doi.org/10.1037/0022-0167.39.3.299

Råbu, M., Binder, P., & Haavind, H. (2013). Negotiating ending: A qualitative study of the process of ending psychotherapy. *European Journal of Psychotherapy & Counselling*, *15*(3), 274–295. https://doi.org/10.1080/13642537.2013.810962

Råbu, M., & Haavind, H. (2012). Coming to an end: A case study of an ambiguous process of ending psychotherapy. *Counselling and Psychotherapy Research*, *12*(2), 109–117. https://doi.org/10.1080/14733145.2011.608131

Råbu, M., & Haavind, H. (2017). Coming to terms: Client subjective experience of ending psychotherapy. *Counselling Psychology Quarterly*, *31*(2), 223–242. https://doi.org/10.1080/09515070.2017.1296410

Råbu, M., Haavind, H., & Binder, P. (2013). We have travelled a long distance and sorted out the mess in the drawers: Metaphors for moving towards the end in psychotherapy. *Counselling and Psychotherapy Research*, *13*(1), 71–80. https://doi.org/10.1080/14733145.2012.711339

Renk, K., & Dinger, T. M. (2002). Reasons for therapy termination in a university psychology clinic. *Journal of Clinical Psychology*, *58*(9), 1173–1181. https://doi.org/10.1002/jclp.10075

Roe, D. (2007). The Timing of Psychodynamically Oriented Psychotherapy Termination and its Relation to Reasons for Termination, Feelings About Termination, and Satisfaction With

Therapy. *The Journal of the American Academy of Psychoanalysis and Dynamic Psychiatry*, *35*(3), 443–453. https://doi.org/10.1521/jaap.2007.35.3.443

Roe, D., Dekel, R., Harel, G., & Fennig, S. (2006a). Clients' reasons for terminating psychotherapy: A quantitative and qualitative inquiry. *Psychology and Psychotherapy: Theory, Research and Practice*, *79*(4), 529–538. https://doi.org/10.1348/147608305x90412

Roe, D., Dekel, R., Harel, G., Fennig, S., & Fennig, S. (2006b). Clients' feelings during termination of psychodynamically oriented psychotherapy. *Bulletin of the Menninger Clinic*, *70*(1), 68–81. https://doi.org/10.1521/bumc.2006.70.1.68

Safarzade, S., Boogar, I. R., & Talepasand, S. (2018). The role of demographical factors in prediction of successful termination of individual psychotherapy. *Journal of Research & Health*, *8*(3), 198–209. https://doi.org/10.29252/jrh.8.3.198

Schen, C. R., Raymond, L., & Notman, M. T. (2013). Transfer of care of psychotherapy patients: Implications for Psychiatry Training. *Psychodynamic Psychiatry*, *41*(4), 575–595. https://doi.org/10.1521/pdps.2013.41.4.575

Schulman, R., & Kay, J. (1989). Transferring patients from residency into practice. *Academic Psychiatry*, *13*(2), 92–95. https://doi.org/10.1007/bf03341266

Shafran, N., Kline, K., Marks, E., Gupta, S., Pinto-Coelho, K. G., Kanazawa, Y., & Hill, C. E. (2019). The final session of psychodynamic psychotherapy for satisfied and unsatisfied clients who initiate the end of treatment. *Counselling Psychology Quarterly*, *33*(4), 583–597. https://doi.org/10.1080/09515070.2019.1635435

Sullivan, D. J., Zeff, P., & Zweig, R. A. (2018). Psychotherapy Termination Practices with Older Adults: Impact of Patient and Therapist Characteristics. *Clinical Gerontologist*, *41*(5), 399–411. https://doi.org/10.1080/07317115.2018.1437101

Todd, D. M., Deane, F. P., & Bragdon, R. A. (2002). Client and therapist reasons for termination: A conceptualization and preliminary validation. *Journal of Clinical Psychology*, *59*(1), 133–147. https://doi.org/10.1002/jclp.10123

Tryon, G. S., & Kane, A. S. (1993). Relationship of working alliance to mutual and unilateral termination. *Journal of Counseling Psychology*, *40*(1), 33–36. https://doi.org/10.1037/0022-0167.40.1.33

Tryon, G. S., & Kane, A. S. (1995). Client involvement, working alliance, and type of therapy termination. *Psychotherapy Research*, *5*(3), 189–198. https://doi.org/10.1080/10503309512331331306

Vaslamatzis, G., Markidis, M., & Katsouyanni, K. (1989). Study of the patients' difficulties in ending brief psychoanalytic psychotherapy. *Psychotherapy and Psychosomatics*, *52*(4), 173–178. https://doi.org/10.1159/000288321

Weil, M. P., Katz, M., & Hilsenroth, M. J. (2017). Patient and therapist perspectives during the psychotherapy termination Process: the role of participation and exploration. *Psychodynamic Psychiatry*, *45*(1), 23–43. https://doi.org/10.1521/pdps.2017.45.1.23

Westmacott, R., & Hunsley, J. (2010). Reasons for terminating psychotherapy: a general population study. *Journal of Clinical Psychology*, *66*(9), 965–977. https://doi.org/10.1002/jclp.20702

Westmacott, R., & Hunsley, J. (2017). Psychologists' Perspectives on Therapy Termination and the Use of Therapy Engagement/Retention Strategies. *Clinical Psychology & Psychotherapy*, *24*(3), 687–696. https://doi.org/10.1002/cpp.2037

Westmacott, R., Hunsley, J., Best, M., Rumstein-McKean, O., & Schindler, D. (2010). Client and therapist views of contextual factors related to termination from psychotherapy: A comparison between unilateral and mutual terminators. *Psychotherapy Research*, *20*(4), 423–435. https://doi.org/10.1080/10503301003645796
